# *Bacillus* benefits the competitive growth of *Ambrosia artemisiifolia* by increasing available nutrient levels

**DOI:** 10.3389/fpls.2022.1069016

**Published:** 2023-01-12

**Authors:** Fengjuan Zhang, Jianru Sun, Chang Wang, Chunying Li, Fengxin Chen, Haiyun Xu, Xue Chen

**Affiliations:** ^1^ College of Life Science, Hebei University, Baoding, Hebei, China; ^2^ School of Life Sciences, Fudan University, Shanghai, China

**Keywords:** *Ambrosia artemisiifolia*, *Bacillus* community, rhizosphere soil, competitive growth, nutrient uptake, *Bacillus megaterium*

## Abstract

*Bacillus* can help plants to acquire nutrients either directly or indirectly. However, the role of *Bacillus* community on the competitive growth of invasive *Ambrosia artemisiifolia* is poorly understood. Native *Setaria viridis* is often found in areas that have been invaded by *A. artemisiifolia*. We sought to determine whether the quantitative and/or qualitative differences in the *Bacillus* community present on the invasive *A. artemisiifolia* and native *S.viridis* provide a competitive advantage to the invasive over native species. A field experiment was established to imitate the invasion of *A. artemisiifolia*. The 16S rRNA gene was commercially sequenced to identify the bacilli isolated from the rhizosphere soil of field-grown *A. artemisiifolia* and *S. viridis*. The *Bacillus* communities in their rhizosphere were compared, and their effects on the competitive growth of *A. artemisiifolia* and *S. viridis* were tested in the pot experiments. *Bacillus* in the rhizosphere soil of *A. artemisiifolia* significantly enhanced its intra-specific competitive ability. The relative abundance of *B. megaterium* in the rhizosphere soil of *A. artemisiifolia* was significantly higher than that of *S. viridis*. Inoculation with *B. megaterium* that was isolated from the rhizosphere soil of both *A. artemisiifolia* and *S. viridis* significantly enhanced the relative competitiveness of *A. artemisiifolia* and inhibited that of *S. viridis*. The higher abundance of *B. megaterium* in the rhizosphere of *A. artemisiifolia* creates higher levels of available nutrients than that in the native *S. viridis*, which enhance the competitive growth of *A. artemisiifolia*. The result helps to discover the mechanism of *Bacillus* community in the invasion of *A. artemisiifolia*.

## 1 Introduction

Determining the comparative impacts of increased intra-versus inter-specific competition is important in the ecosystems for understanding the ecological changes by the invasion of invasive species (De Santis et al., 2021). In general, invasive species often outcompete native species when colonizing new ranges (Bernard-Verdier and Hulme, 2019). The growth and expansion of a plant species in a diverse community of competing organisms depend on two components: its ability to absorb nutrients from the soil and its impact on the available soil resources (Davis et al., 2000; Chase and Leibold, 2003). Some studies have delineated that invasive species are more effective in acquiring nutrients than native species (Pattison et al., 1998; Baruch and Goldstein, 1999; Feng, 2008). Some studies also pointed out that invasive species adapt to different levels of nutrient environments more quickly and are better able to modify resources than the native species, thus, the higher inter-specific competition of the invasive species leads to its successful (Davidson et al., 2011; Parker et al., 2013; Luo et al., 2019). Upon the successful invasion of a new range, the invasive species may exploit unused resources, modify resources to benefit themselves or weaken the growth of other species (Shea and Chesson, 2002; Chase and Leibold, 2003; Parker et al., 2019). Therefore, it is crucial to investigate the potential differences in the modification of resources by invasive and native species during an invasion.

Soil microbial communities are essential in the soil nutrient cycle (Fraterrigo et al., 2006; Iwaoka et al., 2018). The rhizosphere microbiome is involved in important processes, such as nitrogen fixation, the mobilization of phosphorus, and the alteration of other nutrients (Rodríguez-Caballero et al., 2020). Moreover, plants can alter the microbial community in the rhizosphere and may accumulate some beneficial microorganisms in their rhizospheres, such as *Bacillus* spp., arbuscular mycorrhizal fungi (AMF), and *Pseudomonas* spp. (Ehrenfeld, 2003; Kourtev et al., 2003; Gibbons, 2017; Chen et al., 2022). *Bacillus* is one of the rhizosphere-promoting bacterial genera known as a natural plant nutrition resource (Saxena et al., 2019). In addition, members of the genus *Bacillus* are known to have multiple beneficial traits that directly or indirectly aid plants to acquire nutrients (Saxena et al., 2019). Different *Bacillus* spp. varied in their ability to fix nitrogen and solubilize and mineralize phosphorus (Goswami et al., 2016; Pramanik et al., 2019). Moreover, root exudates are among the important factors that influence those abilities (Tariq et al., 2007; Zhang et al., 2014; Shakeel et al., 2015; Wang et al., 2020). The difference between the root exudates of invasive and native species may enable them to recruit specific *Bacillus* species in their rhizosphere and change their ability to fix nitrogen, and solubilize and mineralize phosphorus (Chen et al., 2022). The specific *Bacillus* species in the rhizosphere soil of the invasive plants may help the invading species exploit much more unused resources in the soil and eventually become the dominant plant species (Chen et al., 2022). However, only a few studies have investigated the interaction between invasive species and *Bacillus* species (Dai et al., 2016; Sun et al., 2020). Therefore, understanding the differences in *Bacillus* communities and their function between native and invasive populations would help to predict the ecological roles of *Bacillus* communities during the process of plant invasion.

The invasive species *A. artemisiifolia*, commonly known as ragweed, is a member of the family Asteraceae and is widely distributed in China (Xu and Qiang, 2004). Its invasion poses a serious threat to biodiversity and agricultural production (Ozaslan et al., 2016). In addition, it often causes human health problems owing to its allergenic pollen (Ghiani et al., 2012). *S. viridis* (L.) Beauv. (Poaceae) is an annual C_4_ monocotyledon species native to China. It is often found in areas invaded by *A. artemisiifolia*. According to Zhang et al. (2018), invasion by *A. artemisiifolia* changes the rhizosphere microbial community in its rhizosphere soil. In particular, when *A. artemisiifolia* grows with native *S. viridis*, the increase in AMF colonization in *A. artemisiifolia* and the decrease in *S. viridis* provide a competitive advantage to invasive species over the native species (Zhang et al., 2018). Furthermore, *Bacillus* spp. are often found in the rhizosphere soil of invasive plants (Huangfu et al., 2015; Song et al., 2017). However, the effect of the invasion of *A. artemisiifolia* on *Bacillus* community and the related mechanism is poor understood currently.

To test the role of *Bacillus* spp. in the invasion of *A. artemisiifolia*, we hypothesized that: (i) the difference in *Bacillus* diversity in the rhizosphere soil of between *A. artemisiifolia* and *S. viridis* lead to the different effect on the competitive growth of *A. artemisiifolia*; and (ii) the assembly of specific *Bacillus* isolates increased the competitive advantage of the invasive species more than the native species by providing more available nutrients. We then conducted three sets of experiments to test our hypotheses. Firstly, *Bacillus* was separately isolated from the rhizosphere soil of *A. artemisiifolia* and *S. viridis*, and a comparison of *Bacillus* diversity was performed to determine whether *A. artemisiifolia* recruits specific *Bacillus* species in its rhizosphere soil. Secondly, a comparison of the effect of *Bacillus* isolated from the rhizosphere soil of *A. artemisiifolia* and *S. viridis* on the competitive growth of *A. artemisiifolia* was performed in a greenhouse experiment. Lastly, the role of *B. megaterium*, specific *Bacillus* species recruited in its rhizosphere soil of *A. artemisiifolia*, in the competitive growth of *A. artemisiifolia* was evaluated. This results provide a broader understanding of the functional role of *Bacillus* in promoting the invasion of *A. artemisiifolia*.

## 2 Materials and methods

### 2.1 Experiment I: Comparative analysis of the *Bacillus* diversity in the rhizosphere soil of *A. artemisiifolia* and *S. viridis*

#### 2.1.1 Experimental design

A field experiment was established at the Langfang Experimental Station, Chinese Academy of Agricultural Science (CAAS), Beijing, China (39° 30′ 42′′ N, 116° 36′ 07′′ E). The experimental site has a northern temperate climate. The mean annual rainfall and average temperature in 2013 were 712.8 mm and 11.2 °C, respectively. Our experimental design was described by Zhang et al. (2018). The experimental plots (3 m × 2 m) were prepared in 2008, with a 1 m isolation zone to prevent edge effects (**Figure S1A**). The three treatments that were used in the experiment included: (1) *S. viridis* monoculture (S), (2) an equal mixture of *A. artemisiifolia* and *S. viridis* (A:S=1:1), and (3) *A. artemisiifolia* monoculture (A) (**Figure S1A**).

#### 2.1.2 Soil sampling and *Bacillus* isolation

After 11 years, the abundance of each plant species had changed, so we reduced the number of replicates to three, with similar aerial plant cover. In 2019, the cover of *A. artemisiifolia* and *S. viridis* in the monocultures was 99% and 93%, respectively, and 76% and 20%, respectively, in the mixed treatment. We identified the outliers of plants as described by Huber (2011) and thinned the samples to obtain a sample that would robustly reflect the mean. We visually estimated the mean and chose plants that appeared to be typical in each treatment. Five plants in each treatment were selected per species and pooled together for subsequent index determination. Soil was collected from the rhizosphere of *A. artemisiifolia* and *S. viridis* in each plot (**Figure S1B**) (Zhang et al., 2018). We isolated bacilli from the soil samples as described by Chikerema et al. (2012) with modifications. The procedure was listed in **Figure S1C**.

#### 2.1.3 Analysis of *Bacillus* diversity

We extracted DNA from the colony as described by **Figure S1D** (Chen et al., 2022). The 16S rRNA gene was commercially sequenced to identify the bacilli isolated. The full-length gene was amplified from the bacterial DNA using the universal forward primer F27 (5′-AGAGTTTGATCMTGGCTCAG-3′) and the reverse primer R1492 (5′-ACGGHTACCTTGTTACGACTT-3′) (Malvick et al., 2010). The procedure of 16S rRNA gene amplification was listed in **Figure S1E**. The amplified PCR product (1500 bp) was separated *via* gel electrophoresis on a 1.0% (w/v) agarose gel. The sequencing was performed commercially by General Biosystems (Anhui, China). Phylogenetic analyses were followed **Figure S1F**. The sequences reported in the experiment were deposited in the EzTaxon database (https://www.ezbiocloud.net/) under the accession numbers MW759418-MW759434.

The relative abundance (*RA*) of the bacilli in the entire sample, including non-*Bacillus* DNA amplified by the *Bacillus* primers, was calculated as: *RA*=*A*/*N*×100%, where *A* indicates the number of sequences of one *Bacillus* phylotype, and *N* indicates the total number of sequences.

The Shannon index (*H’*), Simpson index (D), and evenness index (J) were calculated as additional measures of the *Bacillus* diversity (Whittaker, 1972; Hill, 1973). The formulae included:


$$H'=-\sum_{i=1}^{S}p_{i}\ln p_{i}$$

$$p_{i}=n_{i}/N,$$

$$D=1-\sum {\text{i}}^{2}$$

J=H'/lnS

where *S* represents the total number of *Bacillus* phylotypes; *n_i_* represents the number of *Bacillus* phylotype *i*, and *N* represents the number of all *Bacillus* phylotypes.

### 2.2 Experiment II: Comparative analysis of the effect of *Bacillus* from the rhizosphere soil of *A. artemisiifolia* and *S. viridis* on the competitive growth of *A. artemisiifolia*

#### 2.2.1 *Bacillus* inoculation design

The *Bacillus* strains that were isolated from the rhizosphere soil of *A. artemisiifolia* and *S. viridis* were used to determine their effect on competitive advantage. The population count of isolated *Bacillus* was maintained at 10^8^ CFU/mL. All the strains from the rhizosphere soil of *A. artemisiifolia* and *S. viridis* were mixed at 1:1 (v/v), respectively. There were three treatments in the competitive experiment. Each treatment was divided further into three levels (**Figure S2**). The sandy clay was collected near the experimental field site from an open area that had not been covered with vegetation for the previous three years. Vermiculite was obtained from the Baisheng Plant and Flower Co., Ltd., Baoding, China. Basic soil properties included a pH (w/v soil: water = 1:5) of 8.2, organic matter content of 14.29 g/kg, available nitrogen of 52.18 mg/kg, and available phosphorus of 3.4 mg/kg. Seeds of *A. artemisiifolia* and *S. viridis* were sterilized by 75% ethanol for 3 min and washed with distilled water until no alcohol residue was left. The sterilized seeds were planted in pots with 1 kg soil, and the respective bacterial suspensions (10 mL 10^8^ CFU/mL) were added to initiate the experiment. The plants grown under the different treatments were harvested after 90 days. The roots were washed free of soil and then oven-dried at 80°C for 48 h to collect the growth index data. The entire plant, including the aboveground and root biomass, was used to determine the dry biomass. The corrected index of relative competition intensity (CRCI) and InRR were used to quantify the competitive outcome. The corrected index of relative competition intensity was calculated as described by Oksanen et al. (2006). CRCI = arcsine [(X-Y)/max (X, Y)], where X is the average biomass of individual plants grown without competition and Y is the biomass which grown in competition. CRCI<0 would indicate that the intra-specific competition is higher than inter-specific competition. CRCI >0 would indicate that the intra-specific competition is lower than inter-specific competition. The competitive outcome (lnRR) is the log(invasive/native). If the index lnRR is >0, that means that the invasive plants grew larger than the native plants under the mixture treatment.

### 2.3 Experiment III: Comparative analysis of *Bacillus megaterium* from the rhizospheres of *A. artemisiifolia* and *S. viridis* on the competitive growth of *A. artemisiifolia*

#### 2.3.1 Bacillus megaterium inoculation

The *B. megaterium* strains isolated from the rhizospheres of *A. artemisiifolia* and *S. viridis* in Experiment I were used to test for their effect on the competitive growth of *A. artemisiifolia*. The previously frozen (-20°C) *B. megaterium* strains were thawed in a water bath at 30°C for 90 s. The activation of *B. megaterium* was followed **Figure S3**. All the suspensions of *B. megaterium* strains from *A. artemisiifolia* or *S. viridis* were mixed to 1:1 (v/v), respectively. Four densities of *B. megaterium* from *A. artemisiifolia* or *S. viridis* (C0: 0, C1: 5×10^8^ CFU/mL, C2: 15×10^8^ CFU/mL, and C3: 30×10^8^ CFU/mL of *B. megaterium*) were used for the inoculation experiment, respectively. As for the uninoculated treatment (C0), 1 mL of sterile water was added to 100 mL petri dish of beef paste with a peptone medium. Three treatments were used to determine the effects of *B. megaterium* on the competitiveness of *A. artemisiifolia* compared with *S. viridis* (**Figure S3**).

### 2.3.2 Measured indices

#### 2.3.2.1 Plant growth parameters

*Ambrosia artemisiifolia* and *S. viridis* grown in different treatments were harvested 90 days after germination. The soil was washed from the roots, and the plants were oven-dried at 80°C for 48 h to collect the growth index data. The whole plant, including the aboveground and root biomass, was used to determine the dry biomass. The total dry plant weight data of *A. artemisiifolia* and *S. viridis* were recorded. The CRCI was used to quantify the effect. Twenty milligram of samples of *A. artemisiifolia* and *S. viridis* leaves and stems were analyzed to determine the total carbon content. The carbon content was measured using the potassium dichromate-concentrated sulfuric acid (K_2_Cr_2_O_7_-H_2_SO_4_) oxidation method. Dry *A. artemisiifolia* and *S. viridis* matter (2 g each) were digested in a 1:6 mixture of concentrated perchloric (HClO_4_) and nitric acids (HNO_3_) (v/v) to analyze the contents of total nitrogen and phosphorus. The nitrogen content was determined using the micro-Kjeldahl method (Nelson and Sommers, 1972), while the phosphorus content was measured using inductively coupled plasma spectroscopy (Isaac and Johnson, 1983). Each experiment was replicated 10 times.

#### 2.3.2.2 Available nutrients

Soil available phosphorus extracted by 0.5 M NaHCO_3_ was measured by the molybdenum blue method. Soil ammonium ( $\text{N}{\text{H}}_{4}^{+}$ -N) and nitrate ( $\text{N}{\text{O}}_{3}^{-}$ -N) concentrations in extracts were assessed colorimetrically by automated segmented flow analysis (AAIII; BRAN + LUEBBE, GmbH, Norderstedt, Germany) using the salicylate/dichloroisocyanuric acid and cadmium column/sulfanilamide reduction methods, respectively (Shi et al., 2018). Soil available nitrogen (AN) is the sum of ammonium and nitrate nitrogen.

#### 2.3.2.3 Bacillus megaterium density

The density of *B. megaterium* was determined to compare its growth among different treatments at harvest. This was conducted for each fresh soil sample using serial dilution techniques on agar plates with nutrient broth media. In particular, one gram soil sample was collected from the rhizosphere soil and transferred to a tube when the plants were harvested, and 9 mL distilled water was added. The suspension was shaken to homogeneity at 200 rpm for 24 h, and then heated in a hot water bath at 90°C for 10 minutes. After 12 h of incubation, the supernatant was serially diluted from 10^−2^ to 10^−5^, then up to 0.1 mL was pipetted from each aseptic dilution using a flattened micropipette and added to nutrient agar plates. The plates were incubated at 37°C for 12 h. The density of *B. megaterium* was estimated by counting the single colonies. The *Bacillus* concentration was determined based on the number of colonies and colony separation in the 10^−3^ dilution. The CFU/1 g dry weight of the soil (CFU/g DWs) was calculated based on the volume diluted. Each treatment was replicated in triplicate.

### 2.4 Statistical analysis

Before analyses, all data were checked for normality using the Shapiro–Wilk test, to ensure that it met the normality assumption. A one-way analysis of variance (ANOVA) with a Duncan’s test was performed to test the differences in *Bacillus* diversity between the *A. artemisiifolia* and *S. viridis* rhizosphere soils in different treatments, as well as the effect of bacilli isolated from the rhizosphere soil of *A. artemisiifolia* or *S. viridis* on the CRCI of each species. A two-way ANOVA (Duncan’s test) was used to analyze the effect of competition and *Bacillus* on plant growth parameters, concentrations of available nitrogen and phosphorus in the soil, and the density of *Bacillus*. The Tukey’s Honest Significant Difference test was applied to compare the means between treatments. A Pearson’s correlation analysis (two-sided test) was used to determine the relationship between the concentration of *B. megaterium* and plant growth indicators in the soil at harvest. All the analyses were performed using SPSS 19.0 (IBM, Inc., Armonk, NY, USA).

## 3 Results

### 3.1 Experiment I: *Bacillus* diversity in the rhizosphere soil

**Figure 1:**
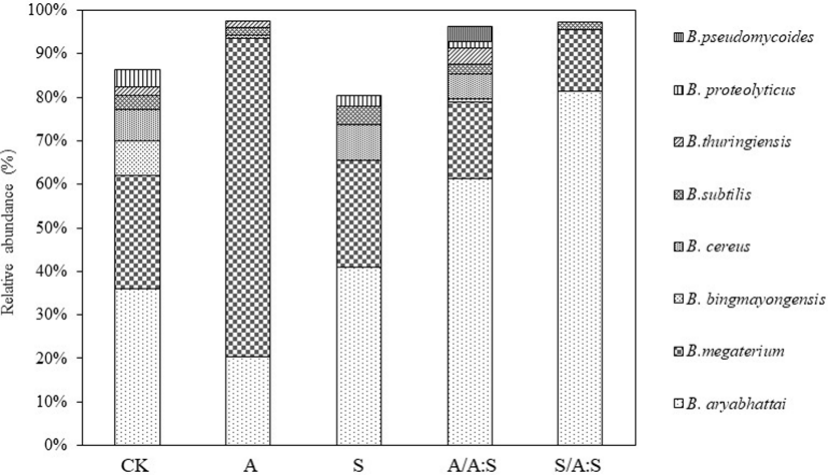
Relative abundance of *Bacillus* species in different treatments soil. C, control; A: *Bacillus* from the rhizosphere soil of *A. artemisiifolia* in the monoculture; S: *Bacillus* from the rhizosphere soil of *A. artemisiifolia* in the monoculture; A/A:S, *Bacillus* from the rhizosphere soil of *A. artemisiifolia* in the mixture; S/A:S, *Bacillus* from the rhizosphere soil of *S. viridis* in the mixture. Error bars represent ±SD of mean (n=3).

A total of 17 species of *Bacillus* were identified in the rhizosphere soil (**Figure S4**). The *Bacillus* diversity varied among the different treatments. For instance, seven and eleven *Bacillus* phylotypes were identified in the rhizosphere soil of *A. artemisiifolia* in A and A:S. The rhizosphere soil of *S. viridis* in S and A:S had 10 and 6 *Bacillus* phylotypes, respectively (**Figure S4**; **Table S1**). The Shannon index, Simpson index, and evenness index of *Bacillus* diversity in the rhizosphere soil of *A. artemisiifolia* in the A treatment were lower than those in the A:S treatment (all, *P*<0.05). While the *Bacillus* diversity indices in the rhizosphere soil of *S. viridis* in the S treatment were higher than those in the A:S treatment, respectively (all, *P*<0.05) (**Table 1**). *Bacillus megaterium* was one of the predominant species in all the soil samples, particularly in the rhizosphere soils of *A. artemisiifolia* in A and S (**Figure 1**).

**Table 1:** The bacillus diversity analysis of different soil.

Samples	Shannon index	Simpson index	Evenness index
CK	1.8 4± 0.08a	0.78 ± 0.01a	0.73 ±0.04a
A	0.82 ± 0.04d	0.42 ± 0.02d	0.43±0.03c
A/A:S	1.31 ± 0.01c	0.58 ± 0.01c	0.61 ± 0.01b
S/A:S	0.62 ± 0.03e	0.31 ± 0.02e	0.38 ±0.04c
S	1.73 ± 0.05b	0.75 ± 0.02b	0.76± 0.02a

Treatments: Data represent Mean ± SD. CK (bare soil), A (A.artemisiifolia monoculture), A/A:S (*A. artemisiifolia* in the mixture of *A. artemisiifolia* and *S. viridis*), S (*S. viridis* monoculture), S/A:S (*S. viridis* in the mixture of *A. artemisiifolia* and *S. viridis*). Different lowercase letters indicate significant differences of the same indicator among different treatments at *P* < 0.05 (n = 3).

### 3.2 Experiment II: A comparative analysis of the effect of *Bacillus* from the rhizosphere soil of *A. artemisiifolia* and *S. viridis* on the competitive growth of *A. artemisiifolia*

**Figure 2:**
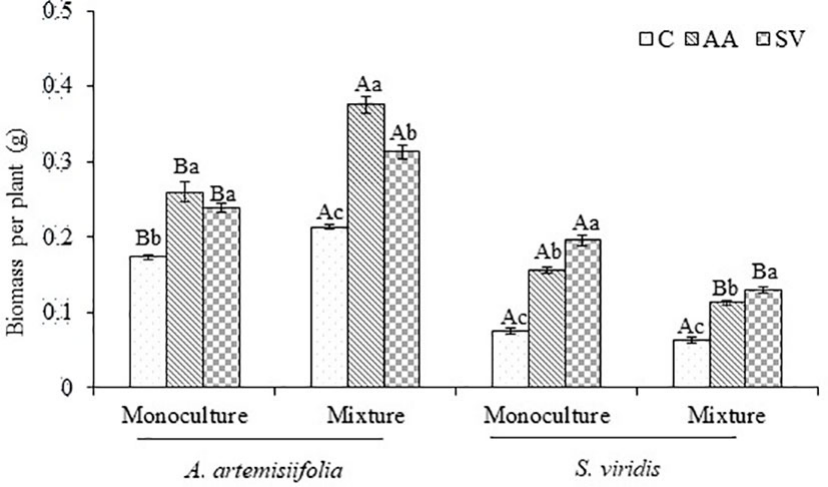
Effects of competition and inoculum on the biomass of *A. artemisiifolia* and *S. viridis*. Means and SDC, control; AA: *Bacillus* from the rhizosphere soil of *A. artemisiifolia* in the monoculture; SV: *Bacillus* from the rhizosphere soil of *S. viridis* in the monoculture. Different uppercase letters indicate significant differences between mixture treatments and monoculture treatments receiving the same microbial inoculations at P < 0.05. Different lowercase letters indicate significant differences among inoculation of different *Bacillus* at *P*<0.05. Error bars represent ±SD of mean (*n*=10).

**Figure 3:**
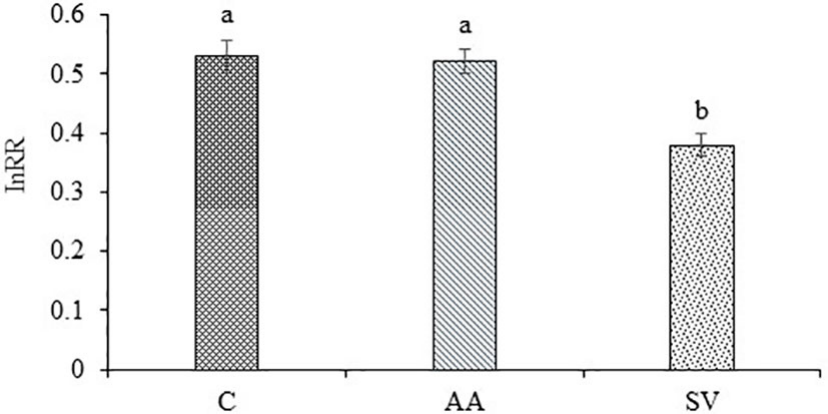
Effects of competition on InRR of *A. artemisiifolia* and *S. viridis*. C, control; AA: *Bacillus* from the rhizosphere soil of *A. artemisiifolia*; SV: *Bacillus* from the rhizosphere soil of *S. viridis*. Different lowercase letters indicate significant differences among inoculation of different *Bacillus* at *P*<0.05. Error bars represent ±SD of mean (*n*=10).

Compared with the monoculture of *A. artemisiifolia*, the total dry biomass of *A. artemisiifolia* in the mixture treatment increased by 44.29% in the uninoculated treatment (*F*=108.495, *P*<0.001) (**Figure 2**). When *Bacillus* from the rhizosphere soil of *A. artemisiifolia* was inoculated, the biomass of *A. artemisiifolia* increased significantly by 75.79% compared with the uninoculated treatment (*F*=99.723, *P*<0.001). The biomass of *A. artemisiifolia* inoculated with *Bacillus* from the *A. artemisiifolia* soil increased by 20.02% compared with that from the *S. viridis* soil (*P*<0.001). The InRR in the treatment of inoculation with *Bacillus* from the rhizosphere soil of *A. artemisiifolia* increased by 37.77% compared with that from *S. viridis* (**Figure 3**). The CRCI also suggested that *Bacillus* in the rhizosphere soil of *A. artemisiifolia* enhanced its competitive growth (**Figure S5**).

The biomass of *S. viridis* inoculated with *Bacillus* from the *A. artemisiifolia* soil or from the *S. viridis* soil was higher than that of the uninoculated treatment. The biomass of *S. viridis* inoculated with *Bacillus* from the *S. viridis* soil increased by 20.37% in the monoculture and 13.53% in the mixture compared with that from *A. artemisiifolia* soil, respectively (**Figure 2**). However, the CRCI of *S. viridis* showed that *Bacillus* in the rhizosphere soil of *S. viridis* decreased its intra-specific competitive growth ability.

### 3.3 Experiment III: Effect of *B. megaterium* on the competitive growth of *A. artemisiifolia*

*Bacillus megaterium* was one of the predominant species in all the soil samples, particularly in the rhizospheres of *A. artemisiifolia* in A and S, their function in the competition between *A. artemisiifolia* and *S. viridis* was compared in the experiment.

### 3.4 Biomass and CRCI

**Figure 4:**
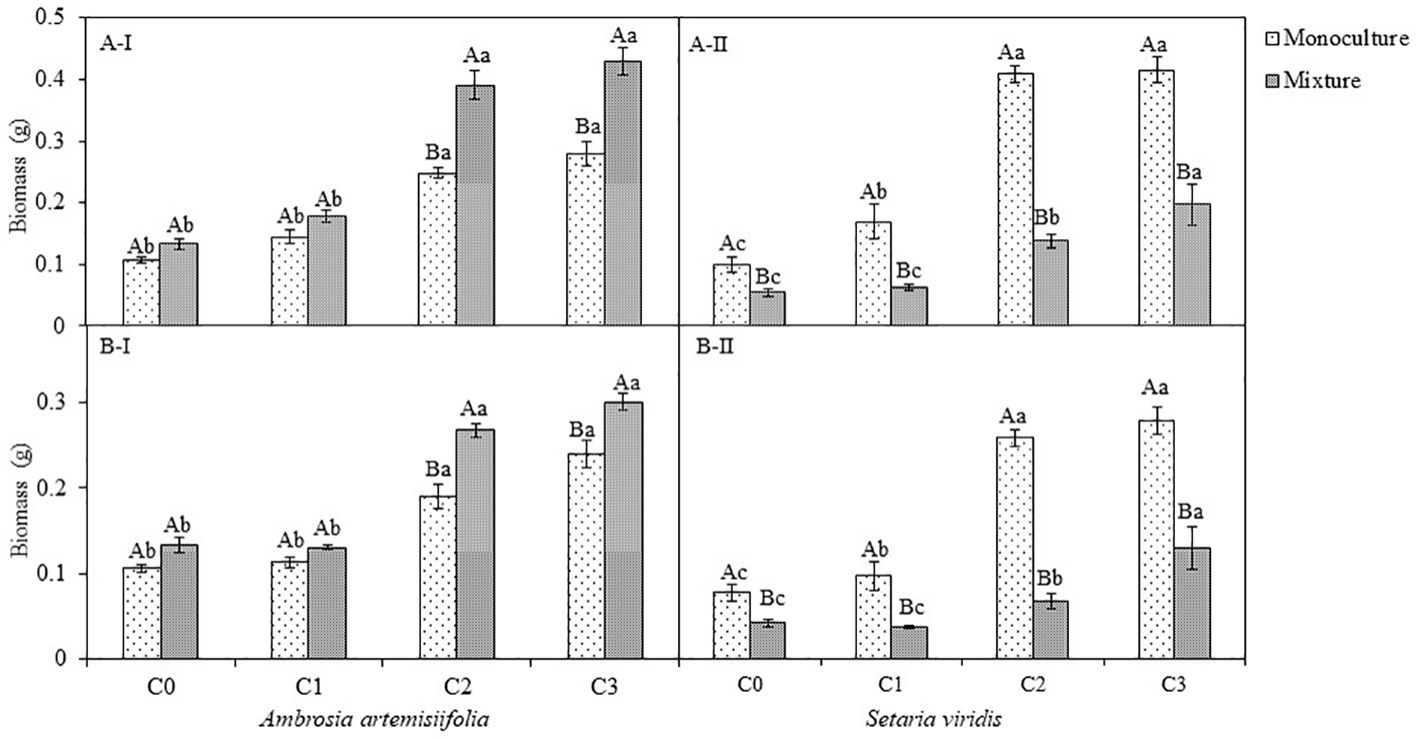
Effects of different concentration of *B megaterium* from the rhizosphere soil of *A artemisiifolia* **(A)** or of *S. viridis* **(B)** on plant biomass, C0, control; C1: 5X 10^8^ cfu/mL of *B. megaterium*; C2: 15 X 10^8^ cfu/mL of *B. megaterium*; C3: 30 X 10^8^ cfu/mL of *B. megaterium*. A-I and A-II represent the biomass of *A artemisiifolia* and *S. viridis* when *B megaterium* from the rhizosphere soil of *A artemisiifolia* was inoculated respectively. B-I and B-II represent the biomass of *A artemisiifolia* and *S. viridis* when *B megaterium* from the rhizosphere soil of *S. viridis* was inoculated respectively. Different uppercase letters indicate significant differences between in the mixture and in the monoculture treatments at *P*<0.05. Different lowercase letters indicate significant differences among different concentrations of *B megaterium* at *P*<0.05. Error bars represent SD of mean (*n*=10).

There was no significant change in the biomass of *A. artemisiifolia* between the monoculture and mixed treatment (with *S. viridis)* (*P*=0.228). Conversely, the competition with *A. artemisiifolia* decreased the biomass of *S. viridis* in the uninoculated (control) treatments (F=136.15; *P*<0.001) (**Figure 4**; **Table S2**). As for the inoculation of *B. megaterium* from *A. artemisiifolia* or *S. viridis*, the biomass in both plant species increased in parallel with the density of *B. megaterium* (*P*<0.05), showing a dose-dependent relationship. Our analysis showed that the biomass of *A. artemisiifolia* in the C2 and C3 inoculation treatments increased significantly by 133.94%-163.20% compared with the uninoculated treatment of the monoculture (all, *P*<0.001). Compared with the monoculture, competition had different effects on the biomass of *A. artemisiifolia* and *S. viridis* in the inoculation with C2 and C3 *B. megaterium*. The biomass of *A. artemisiifolia* increased by 57.59%-53.84%, while that of *S. viridis* decreased by 52.66% (C3)-66.03% (C2) compared with that in the monoculture treatment. The CRCI results further showed that inoculation with C2 and C3 *B. megaterium* concentrations from *A. artemisiifolia* or *S. viridis* enhanced the intra-specific competitive growth ability of *A. artemisiifolia*, while decreasing that of *S. viridis* (**Figure S6**, **Table S2**). The CRCI of *A. artemisiifolia* in the inoculation of *B. megaterium* (C2 and C3) from *A. artemisiifolia* increased by 44.60% (C2) and 61.13% (C3) compared with that from *S. viridis*.

### 3.5 Total carbon, nitrogen, and phosphorus concentrations of *A. artemisiifolia* and *S. viridis*

**Figure 5:**
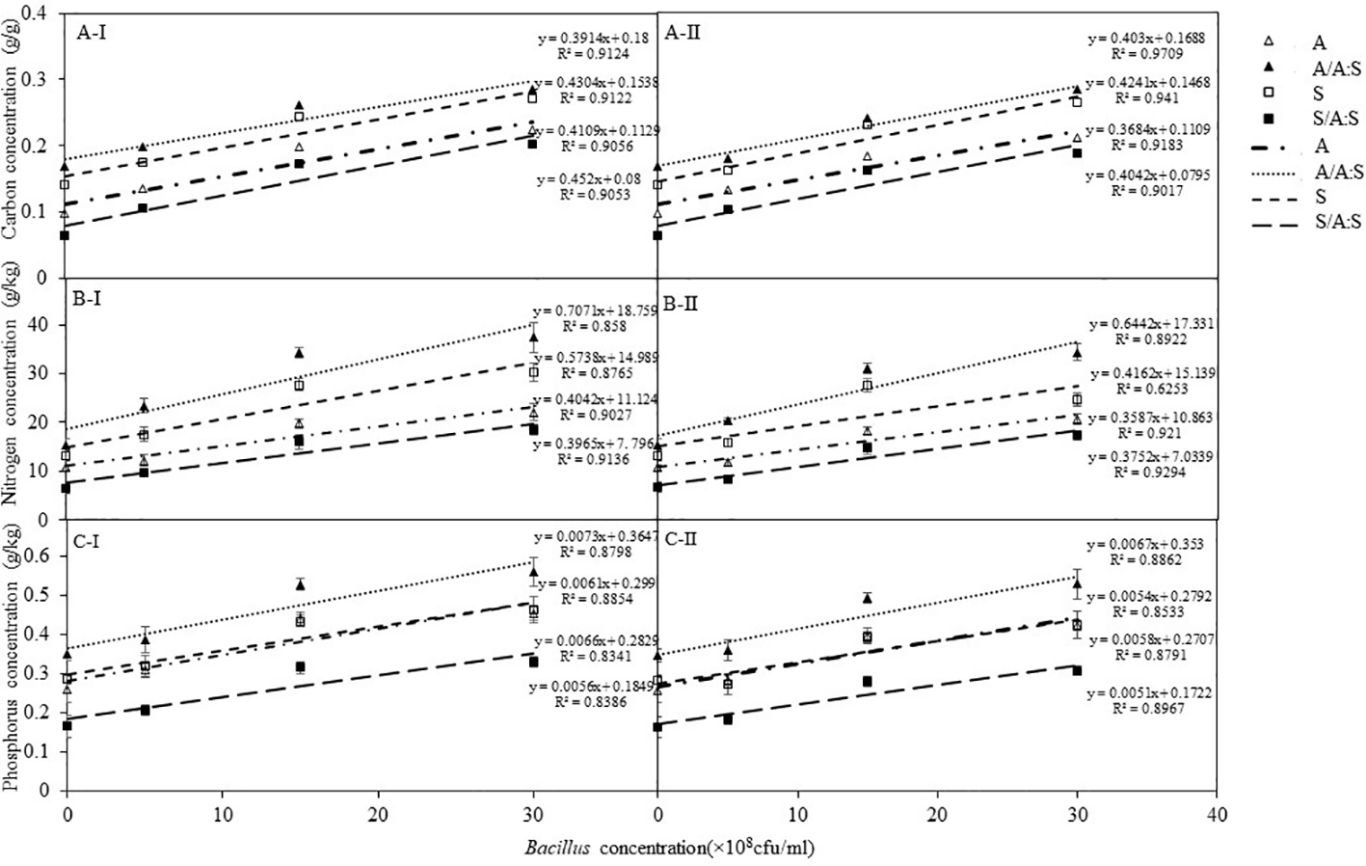
Effects of different concentration of *B megaterium* from the rhizosphere soil of *A artemisiifolia* (I) or of *S. viridis* (II) on carbon **(A)**, nitrogen **(B)** and phosphorus **(C)** levels. of Control; 0; C1: 5X10^8^ cfu/mL of *B. megaterium*; C2: 15X10^8^ cfu/mL of *B. megaterium*; C3: 30 X 10^8^ cfu/mL of *B. megaterium*.

Whether *B. megaterium* was inoculated from *A. artemisiifolia* or *S. viridis*, the bacteria had a dose-dependent effect on the total carbon, nitrogen, and phosphorus concentrations of *A. artemisiifolia* and *S. viridis* (**Figure 5**; **Table S2-S3**). There were no significant differences between the C0 and C1 treatments. In the monoculture of both the C2 and C3 treatments, the total carbon, nitrogen, and phosphorus concentrations of *A. artemisiifolia* or *S. viridis* were significantly higher than in the C0 treatment, respectively. Compared with the monoculture, the total carbon, nitrogen, and phosphorus concentrations of *A. artemisiifolia* in the mixture increased. However, the concentrations in *S. viridis* in the mixture decreased in both the uninoculated and the inoculated treatments (**Table S2-3**).

### 3.6 Available nitrogen and phosphorus concentrations in the soil of different treatments

**Figure 6:**
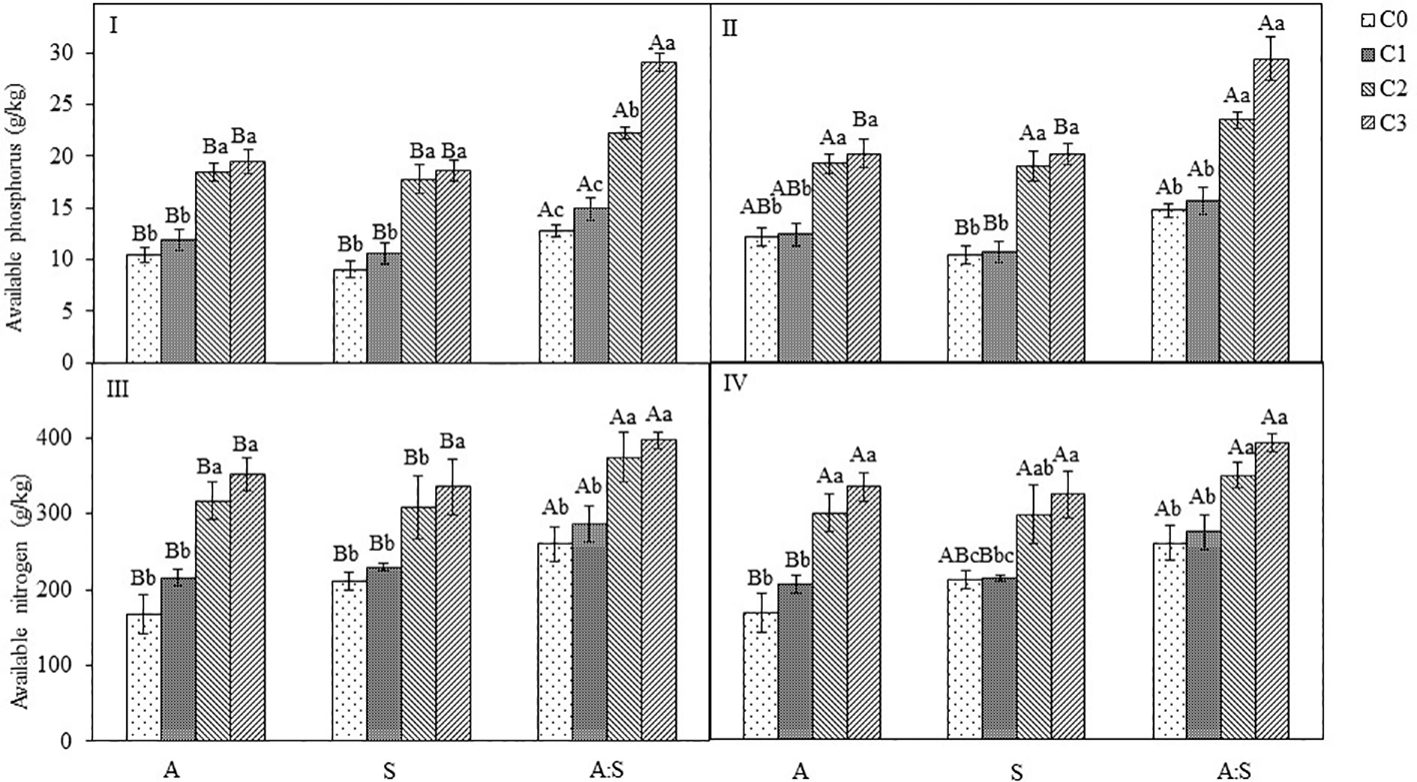
Effects of different concentration of *B megaterium* from the rhizosphere soil of *A artemisiifolia* (I and III) and *S. viridis* (II and IV) on soil available phosphorus and nitrogen levels. C0, control; C1: 5X10^8^ cfu/mL of *B. megaterium*; C2: 15X10^8^ cfu/mL of *B. megaterium*; C3: 30×10^8^ cfu/mL of *B. megaterium*. Different uppercase letters indicate significant differences between in the mixture and in the monoculture treatments at *P* < 0.05. Different lowercase letters indicate significant differences among different concentrations of *B megaterium* at *P*<0.05. Error bars represent ±SD of mean(*n*=10).

There appeared to be no significant difference in available nitrogen and phosphorus concentrations in the soil between C0 and C1 treatments (**Figure 6**; **Table S2-S3**). However, in both C2 and C3 treatments, the total concentrations of carbon, nitrogen and phosphorus of *A. artemisiifolia* or *S. viridis* were significantly higher than those in C0 treatment. The concentrations of total carbon, nitrogen, and phosphorus of *A. artemisiifolia* in the mixture increased compared with those of the monoculture, while the concentrations of *S. viridis* decreased in both the uninoculated and the inoculated treatments (**Figure 6**; **Table S2-S3**).

### 3.7 The density of *B. megaterium* in different treatments

**Figure 7:**
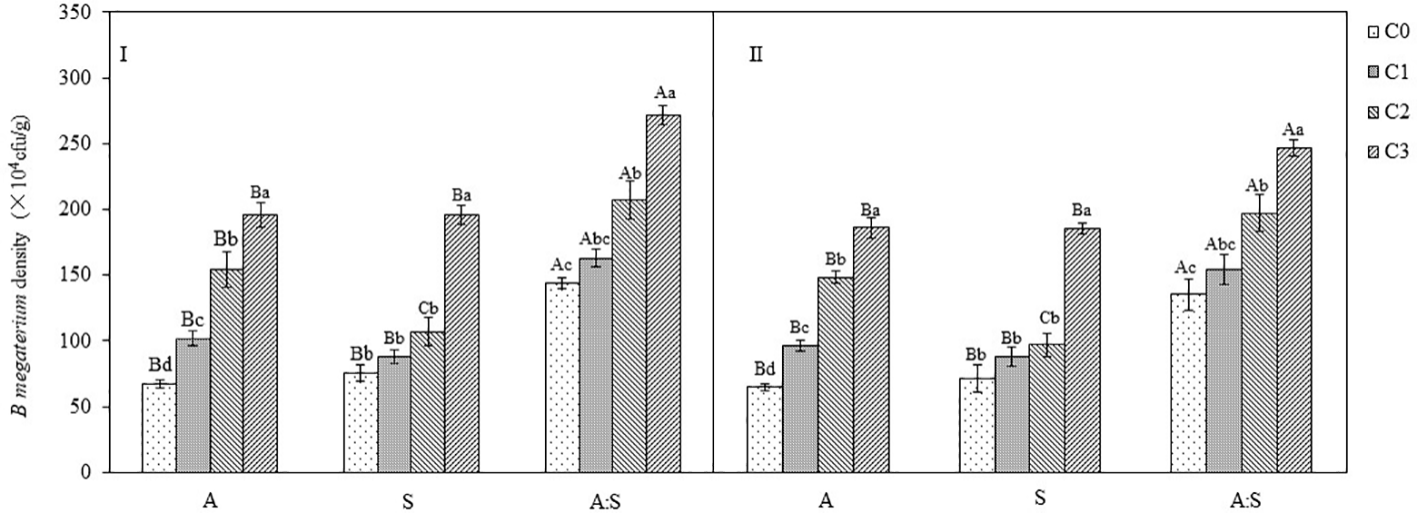
Effects of different concentration of *B megaterium* from the rhizosphere soil of *A artemisiifolia* (I) and *S. viridis* (II) on *B megaterium* density. C0, control; C1: 5×10^8^ cfu/mL of *B. megaterium*; C2: 15×10^8^ cfu/mLof *B. megaterium*; C3: 30×10^8^ cfu/mL of *B. megaterium*. Different uppercase letters indicate significant differences between in the mixture and in the monoculture treatments at *P*<0.05. Different lowercase letters indicate significant differences among different concentrations of *B megaterium* at *P*<0.05. Error bars represent ±SD of mean(*n*=10).

The inoculation of *B. megaterium* from *A. artemisiifolia* or *S. viridis* resulted in a dose-dependent increase in soil *B. megaterium* levels when grown in monoculture (A or S) or mixture (A: S). For the *A. artemisiifolia* monoculture, the density of *B. megaterium* in the C2 and C3 treatments was significantly higher than those in the C0 treatment (*B. megaterium* from *A. artemisiifolia*: *F*=25.306–30.904, *P*=0.001; *B. megaterium* from *S. viridis*: *F*=133.802–182.161, *P*<0.001). It was also found that the density of *B. megaterium* in the soil of mixture treatment was significantly higher than that in the monocultures of *A. artemisiifolia* and *S. viridis* following inoculation with different concentrations of *B. megaterium*, respectively, *B. megaterium* from *A. artemisiifolia*: *F*=49.849, *P*<0.001; *B. megaterium* from *S. viridis*: *F* = 13.436, *P*=0.001; *B. megaterium* from *A. artemisiifolia*: *F*=47.811, *P*<0.001; *B. megaterium* from *S. viridis*: *F*=12.637, *P*=0.001) (**Figure 7**).

### 3.8 Correlation of the density of *B. megaterium* with plant growth indicators

When *B. megaterium* was inoculated from the rhizospheres of *A. artemisiifolia*, its density positively correlated with the growth indicators of *A. artemisiifolia* in all the treatments (**Table S4**). In *S. viridis*, the density of *B. megaterium* positively correlated with the biomass, total carbon, nitrogen, and phosphorus contents in the monoculture, but it was not correlated with the growth indicators of *S. viridis* in the mixture.

When *B. megaterium* was inoculated from the rhizospheres of *S. viridis*, the density of *B. megaterium* positively correlated with the biomass and total carbon of *A. artemisiifolia* in the C0 and C1 treatments. However, it was not correlated with the biomass, total carbon in C3 treatment in both the monoculture and mixture treatments. In *S. viridis*, the density of *B. megaterium* positively correlated with the growth indicators of *S. viridis* in the monoculture. Moreover, the density of *B. megaterium* positively correlated with the biomass, contents of nitrogen, and phosphorus in the C2 and C3 mixture treatments (**Table S5**).

## 4 Discussion

Inter-specific interactions are key factors in the structuring and functioning of ecological communities (Mofu et al., 2019). The difference in plant traits and the rhizosphere soil microbe play key role in the inter-specific interactions. On the one hand, invasive plant has higher inter-specific competition for their competitive advantages over neighboring native species through resource competition (Daehler, 2003; González et al., 2010; Sardans et al., 2017a). On the other hand, invasive plants always modify soil microbe to benefit their own fitness over native species, and then to enhance its inter-specific interaction (Huangfu et al., 2019). In the study, *A. artemisiifolia* and *S. viridis* have different carbon fixation and nutrient absorption abilities, *A. artemisiifolia* assembles some *Bacillus* species (eg. *B. megaterium*) to create higher levels of the available nutrients in its rhizosphere soil than the native *S. viridis*, by promoting its nitrogen and phosphorus absorption to enhance its competitive growth.

Plant functional traits is related with carbon fixation and nutrient absorption abilities, which shapes plant growth (Weidenhamer and Callaway, 2010; Choudhury et al., 2022; Zhang et al., 2022). An increasing body of research shows that invasive plants generally have quite different functional traits from native species, such as higher specific leaf area, leaf area index and photosynthetic rate, which is related to invasion success (Suding et al., 2004; te Beest et al., 2015). *Ambrosia artemisiifolia* and *S. viridis* are members of the Asteraceae and Poaceae families, respectively, and they have different carbon fixation and nutrient absorption abilities. When *A. artemisiifolia* grew alongside *S. viridis* whether in the uninoculated (control) treatments or the inoculated treatments, the competition promoted the growth of *A. artemisiifolia* by increasing its photosynthetic products and absorption of nitrogen and phosphorus. However, the growth of *S. viridis* is inhibited by a reduction in the photosynthetic products and absorption of nitrogen and phosphorus. The different abilities to synthetic photosynthetic product and absorb nutrients leads to their difference in inter-specific competitive ability in the uninoculated (control) treatments, suggesting the successful invasion of *A. artemisiifolia* in resident community is mainly driven by high inter-specific competition.

Soil microbial communities in the rhizosphere have a wide spectrum of plant interactions (Berendsen et al., 2012; Du et al., 2020). The novel weapons hypothesis posits that invasive plants can release secondary compounds into the soil environment from their litter, leaf leachate and root exudate (Zhu et al., 2017), which would, in turn, alters soil microbial community (Lorenzo et al., 2013). The difference in secondary compounds between invasive and native plants may lead to the difference in soil microbial community (Zhu et al., 2017). The difference in *Bacillus* diversity between *A. artemisiifolia* and *S. viridis* further indicates that different functional traits play great roles in shaping microbial community of rhizosphere soil. Besides, increasing evidences show that invasiveness of the invasive species is associated with significant changes in the plant–soil elemental composition and stoichiometry (González et al., 2010; Wu et al., 2019). One of the invasion hypotheses proposes that changes in the soil microbial communities caused by invasive plants can result in positive plant-soil feedback by accumulating beneficial microorganisms in the rhizosphere (Inderjit and Cahill, 2015). *Bacillus* species are prominent soil inhabitants that increase plant growth by various mechanisms, such as the production of growth-stimulating phytohormones, nitrogen fixation, and phosphorus solubilization and mobilization (Wang et al., 2020). The different *Bacillus* diversity between the rhizosphere soil of *A. artemisiifolia* in A and the rhizosphere soil of *S. viridis* in S may lead to their difference in changing soil nutrient cycle. Our further study shows that the different ability of *B. megaterium* to modify soil available nutrients from between the rhizosphere soil of *A. artemisiifolia* in A and the rhizosphere soil of *S. viridis* in S is demonstrated in a *B. megaterium* inoculation experiment. We find that resource modification by plants may be self-beneficial during the inter-specific interactions. Our result indicates that specific *Bacillus* species are gathered in the rhizosphere soils of *A. artemisiifolia* and *S. viridis* during their growth and they differs significantly. Specifically, *Bacillus* from the rhizosphere soil of *A. artemisiifolia* benefited *A. artemisiifolia* more than *S. viridis*, while those from *S. viridis* benefited *S. viridis* more than *A. artemisiifolia*, suggesting both invasive and native species accumulate the different *Bacillus* to benefits its growth. The ability to modify growth-limiting resources is directly related to plant competitive ability (Harpole, 2006; Dybzinski and Tilman, 2007). However, some research show that invasive species can increase the concentrations of available nutrients during their invasion (Sardans et al., 2017b; Zhou and Staver, 2019). For example, Sardans et al. (2017b) reported that the concentrations of soluble nitrate and Olsen-P in soils under invasive plants were 117% and 21% higher, respectively, than in soils under native plant communities. Increasing evidences show that invasiveness of the invasive species is associated with significant changes in the plant–soil elemental composition and stoichiometry (Daehler, 2003; Funk and Vitousek, 2007; González et al., 2010). One of the invasion hypotheses proposes that changes in the soil microbial communities caused by invasive plants can result in positive plant-soil feedback by accumulating beneficial microorganisms in the rhizosphere (Inderjit and Cahill, 2015). The different *Bacillus* diversity the rhizosphere soil of *A. artemisiifolia* in A and the rhizosphere soil of *S. viridis* in S may lead to their difference in changing soil nutrient cycle. Our further study shows that the different ability of *B. megaterium* to modify soil available nutrients from the rhizosphere soil of *A. artemisiifolia* in A and the rhizosphere soil of *S. viridis* in S is demonstrated in a *B. megaterium* inoculation experiment. The increase in available nitrogen and phosphorus concentrations in the soil and their increase in the plants suggests that *B. megaterium* inoculation increases the concentration of available nutrients in the soil. Furthermore, the concentrations of available nitrogen and phosphorus increases in parallel with the density of *B. megaterium*. The density of *B. megaterium* in the rhizosphere soil of *A. artemisiifolia* is higher than that in the rhizosphere soil of *S. viridis*. We hypothesize that *A. artemisiifolia* assembles more *B. megaterium* than native *S. viridis* to increase the level of available nutrients in its rhizosphere soil.

The invasion success of *A. artemisiifolia* in resident community is mainly driven by high inter-specific competition, *B. megaterium* in its rhizosphere soil can modify the resources and enhance its inter-specific competitive growth ability. In the study the effect of *B. megaterium* on the inter-specific competitive growth ability of invasive and native species differed. Compared with the control, *Bacillus* isolated from the rhizosphere soil of both invasive and native species enhanced the inter-specific competitive growth ability of *A. artemisiifolia*, while *Bacillus* from the rhizosphere soil of both invasive and native species decreased the inter-specific competitive growth ability of *S. viridis*, suggesting that *A. artemisiifolia* assembles *B. megaterium* to create higher levels of the available nutrients in its rhizosphere soil than the native *S. viridis*, and then to enhance its competitive growth during its invasion.

## 5 Conclusion

The successful invasion of *A. artemisiifolia* in resident community is mainly driven by high inter-specific competition. *A. artemisiifolia* assembles *B. megaterium* to create higher levels of the available nutrients in its rhizosphere soil than the native *S. viridis*, by promoting its nitrogen and phosphorus absorption to enhance its competitive growth during its invasion.

## Data availability statement

The datasets presented in this study can be found in online repositories. The names of the repository/repositories and accession number(s) can be found in the article/**Supplementary Material**.

## Author contributions

All authors contributed to the designed, writing, and revision of this manuscript and made intellectual contributions.

## References

[B1] Baruch Z. Goldstein G. (1999). Leaf construction cost, nutrient concentration, and net CO_2_ assimilation of native and invasive species in Hawaii. Oecologia 121, 183–192. doi: 10.1007/s004420050920 28308558

[B2] Berendsen R. Pieterse C. Bakker P. A. H. M. (2012). The rhizosphere microbiome and plant health. Trends Plant Sci. 17, 478–486. doi: 10.1016/j.tplants.2012.04.001 22564542

[B3] Bernard-Verdier M. Hulme P. E. (2019). Alien plants can be associated with a decrease in local and regional native richness even when at low abundance. J. Ecol. 107, 1343–1354. doi: 10.1111/1365-2745.13124

[B4] Chase M. Leibold M. A. (2003). Ecological niches: linking classical and contemporary approaches (Chicago: Chicago University Press).

[B5] Chen X. Li Q. Wang Y. Chen F. X. Zhang X. Y. Zhang F. J. (2022). *Bacillus* promotes invasiveness of exotic *Flaveria bidentis* by increasing its nitrogen and phosphorus uptake. J. Plant Ecol. 15, 596–609. doi: 10.1093/jpe/rtab046

[B6] Chikerema S. M. Pfukenyi D. M. Hang’ombe B. M. L’Abee-Lund T. M. Matope G. (2012). Isolation of *Bacillus anthracis* from soil in selected high-risk areas of Zimbabwe. Appl. Environ. Microb. 113, 1389–1395. doi: 10.1111/jam.12006 22984812

[B7] Choudhury M. I. Hallin S. Ecke F. Hubalek V. Juhanson J. Frainer A.. (2022). Disentangling the roles of plant functional diversity and plaint traits in regulating plant nitrogen accumulation and denitrification in freshwaters. Funct. Ecol. 36, 921–932. doi: 10.1111/1365-2435.14001

[B8] Daehler C. (2003). Performance comparisons of co-occurring native and alien invasive plants: implications for conservation and restoration. Annu. Rev. Ecol. Evol. Syst. 34, 183–211. doi: 10.1146/annurev.ecolsys.34.011802.132403

[B9] Dai Z. C. Fu W. Wan L. Y. Cai H. H. Wang N. Qi S. S.. (2016). Different growth promoting effects of endophytic bacteria on invasive and native clonal plants. Front. Plant Sci. 7. doi: 10.3389/fpls.2016.00706 PMC4878316 27252722

[B10] Davidson A. M. Jennions M. Nicotra A. B. (2011). Do invasive species show higher phenotypic plasticity than native species and if so, is it adaptive? A meta-analysis Ecol. Lett. 14, 419–431. doi: 10.1111/j.1461-0248.2011.01596.x 21314880

[B11] Davis M. A. Grime J. P. Thompson K. A. (2000). Fluctuating resources in plant communities: A general theory of invasibility. J. Ecol. 88, 528–534. doi: 10.1046/j.1365-2745.2000.00473.x

[B12] De Santis V. Roberts C. G. Britton R. (2021). Trophic consequences of competitive interactions in freshwater fish: Density dependent effects and impacts of inter-specific versus intra-specific competition. Freshw. Biol. 66, 362–373. doi: 10.1111/fwb.13643

[B13] Du E. W. Chen X. Li Q. Chen F. X. Xu H. Y. Zhang F. J. (2020). *Rhizoglomus intraradices* and associated *Brevibacterium frigoritolerans* enhance the competitive growth of *Flaveria bidentis* . Plant soil 453, 281–295. doi: 10.1007/s11104-020-04594-1

[B14] Dybzinski R. Tilman D. (2007). Resource use patterns predict long-term outcomes of plant competition for nutrients and light. Am. Nat. 170, 305–318. doi: 10.1086/519857 17879183

[B15] Ehrenfeld J. G. (2003). Effects of exotic plant invasions on soil nutrient cycling processes. Ecosystems 6, 503–523. doi: 10.1007/s10021-002-0151-3

[B16] Feng Y. L. (2008). Photosynthesis, nitrogen allocation and specific leaf area in invasive *Eupatorium adenophorum* and native *Eupatorium japonicum* grown at different irradiances. Physiol. Plant 133, 318–326. doi: 10.1111/j.1399-3054.2008.01072.x 18312498

[B17] Fraterrigo J. M. Balser T. C. Turner M. G. (2006). Microbial community variation and its relationship with nitrogen mineralization in historically altered forests. Ecology 87, 570–579. doi: 10.1890/05-0638 16602287

[B18] Funk J. L. Vitousek P. M. (2007). Resource-use efficiency and plant invasions in low-resource systems. Nature 446, 1079–1081. doi: 10.1038/nature05719 17460672

[B19] Ghiani A. Aina R. Asero R. Bellotto E. Citterio S. (2012). Ragweed pollen collected along high-traffic roads shows a higher allergenicity than pollen sampled in vegetated areas. Allergy 67, 887–894. doi: 10.1111/j.1398-9995.2012.02846.x 22582710

[B20] Gibbons S. M. (2017). Microbial community ecology: Function over phylogeny. Nat. Ecol. Evol. 1, 32. doi: 10.1038/s41559-016-0032 28812558

[B21] González A. L. Kominoski J. S. Danger M. Ishida S. Iwai N. Rubach A. (2010). Can ecological stoichiometry help explain patterns of biological invasions? Oikos 119, 779–790. doi: 10.1111/j.1600-0706.2009.18549.x

[B22] Goswami D. Thakker J. N. Dhandhukia P. C. Tejada M. M. (2016). Portraying mechanics of plant growth promoting rhizobacteria (PGPR): a review. Cogent Food Agric. 2, 1127500. doi: 10.1080/23311932.2015.1127500

[B23] Harpole W. (2006). Resource-ratio theory and the control of invasive plants. Plant Soil 280, 23–27. doi: 10.1007/s11104-005-2951-7

[B24] Hill M. O. (1973). Diversity and evenness: a unifying notation and its consequences. Ecology 54, 427–432. doi: 10.2307/1934352

[B25] Huangfu C. H. Hui D. F. Qi X. X. Li K. L. (2019). Plant interactions modulate root litter decomposition and negative plant-soil feedback with an invasive plant. Plant Soil 437, 179–194. doi: 10.1007/s11104-019-03973-7

[B26] Huangfu C. H. Li H. Y. Chen X. W. Liu H. M. Yang D. L. (2015). The effects of exotic weed *Flaveria bidentis* with different invasion stages on soil bacterial community structures. Afr J. Biotechnol. 14, 2636–2643. doi: 10.5897/AJB2015.14658

[B27] Huber P. J. (2011). “Robust statistics,” in International encyclopedia of statistical science, vol. 120 . Ed. Lovric M. (Berlin: Springer), 1–80. doi: 10.1007/978-3-642-04898-2

[B28] Inderjit Cahill J. F. (2015). Linkages of plant–soil feedbacks and underlying invasion mechanisms. AoB Plants. 7, 1-8. doi: 10.1093/aobpla/plv022 PMC4404623 25784668

[B29] Isaac R. A. Johnson W. C. (1983). High speed analysis of agriculture samples using inductively coupled plasma-atomic emission spectroscopy. Spectrochim Acta Part B. 38, 277–282. doi: 10.1016/0584-8547(83)80124-4

[B30] Iwaoka C. Imada S. Taniguchi T. Du S. Yamanaka N. Tateno R. (2018). The impacts of soil fertility and salinity on soil nitrogen dynamics mediated by the soil microbial community beneath the halophytic shrub tamarisk. Microb. Ecol. 75, 985–996. doi: 10.1007/s00248-017-1090-z 29032430

[B31] Kourtev P. S. Ehrenfeld J. G. Haggblom M. (2003). Experimental analysis of the effect of exotic and native plant species on the structure and function of soil microbial communities. Soil Biol. Biochem. 35, 895–905. doi: 10.1016/S0038-0717(03)00120-2

[B32] Lorenzo P. Pereira C. S. Rodríguez-Echeverría S. (2013). Differential impact on soil microbes of allelopathic compounds released by the invasive Acacia dealbata Link. Soil Biol. Biochem 57, 157–163. doi: 10.1016/j.soilbio.2012.08.018

[B33] Luo X. Xu X. Y. Zheng Y. Guo H. Hu S. J. (2019). The role of phenotypic plasticity and rapid adaptation in determining invasion success of *Plantago virginica* . Biol. Invasions 21, 2679–2692. doi: 10.1007/s10530-019-02004-x

[B34] Malvick D. Syverson R. Mollov D. Ishimaru C. A. (2010). Goss's bacterial blight and wilt of corn caused by *Clavibacter michiganensis* subsp. nebraskensis occurs in Minnesota. Plant Dis. 94, 1064. doi: 10.1094/PDIS-94-8-1064A 30743471

[B35] Mofu L. South J. Wasserman R. J. Dalu T. Woodford D. J. Dick J. T. A.. (2019). Inter-specific differences in invader and native fish functional responses illustrate neutral effects on prey but superior invader competitive ability. Freshw. Biol. 64, 1655–1663l. doi: 10.1111/fwb.13361

[B36] Nelson D. W. Sommers L. E. (1972). Determination of total nitrogen in plant material. Agron. J. 65, 109–112. doi: 10.2134/agronj1973.00021962006500010033x

[B37] Oksanen L. Sammul M. Magi M. (2006). On the indices of plant-plant competition and their pitfalls. Oikos 112, 149–155. doi: 10.1111/j.0030-1299.2006.13379.x

[B38] Ozaslan C. Onen H. Farooq S. Gunal H. Akyol N. (2016). Common ragweed: An emerging threat for sunflower production and human health in Turkey. Weed Biol. Manage. 16, 42–55. doi: 10.1111/wbm.12093

[B39] Parker S. S. Harpole W. S. Seabloom E. W. (2019). Plant species natural abundances are determined by their growth and modification of soil resources in monoculture. Plant Soil 445, 273–287. doi: 10.1007/s11104-019-04299-0

[B40] Parker J. D. Torchin M. E. Hufbauer R. A. Lemoine N. P. Alba C. Blumenthal D. M.. (2013). Do invasive species perform better in their new ranges? Ecology 94, 985–994. doi: 10.1890/12-1810.1 23858639

[B41] Pattison R. R. Goldstein G. Ares A. (1998). Growth, biomass allocation and photosynthesis of invasive and native Hawaiian rain-forest species. Oecologia 117, 449–459. doi: 10.1007/s004420050680 28307669

[B42] Pramanik P. Goswami A. J. Ghosh S. Kalita C. (2019). An indigenous strain of potassium-solubilizing bacteria *Bacillus pseudomycoides* enhanced potassium uptake in tea plants by increasing potassium availability in the mica waste-treated soil of north-east India. J. Appl. Microbiol. 126, 215–222. doi: 10.1111/jam.14130 30326179

[B43] Rodríguez-Caballero G. Caravaca F. Diaz G. Torres P. Roldan A. (2020). The invader *Carpobrotus edulis* promotes a specific rhizosphere microbiome across globally distributed coastal ecosystems. Sci. Total Environ. 719, 137347. doi: 10.1016/j.scitotenv.2020.137347 32120096

[B44] Sardans J. Alonso R. Janssens I. A. Carnicer J. Vereseglou S. Rillig M. C.. (2017b). Foliar and soil concentrations and stoichiometry of nitrogen and phosphorous across European *Pinus sylvestris* forests: relationships with climate, n deposition and tree growth. Funct. Ecol. 30, 676–689. doi: 10.1111/1365-2435.12541

[B45] Sardans J. Bartrons M. Margalef O. Gargallo-Garriga A. Janssens I. A. Ciais P.. (2017a). Plant invasion is associated with higher plant–soil nutrient concentrations in nutrient-poor environments. Global Change Biol. 23, 1282–1291. doi: 10.1111/gcb.13384 27272953

[B46] Saxena A. K. Kumar M. Chakdar H. Anuroopa N. Bagyaraj D. J. (2019). *Bacillus* species in soil as a natural resource for plant health and nutrition. J. Appl. Microbiol. 128, 1583–1594. doi: 10.1111/jam.14506 31705597

[B47] Shakeel M. Rais A. Hassan M. N. Hafeez F. Y. (2015). Root associated bacillus sp. improves growth, yield and zinc translocation for basmati Rice(*Oryza sativa*) varieties. Front. Microbiol. 6. doi: 10.3389/fmicb.2015.01286 PMC4649038 26635754

[B48] Shea K. Chesson P. (2002). Community ecology theory as a framework for biological invasions. Trends Ecol. Evol. 17, 170–176. doi: 10.1016/s0169-5347(02)02495-3

[B49] Shi Y. Li Y. T. Xiang X. J. Sun R. B. Yang T. He D.. (2018). Spatial scale affects the relative role of stochasticity versus determinism in soil bacterial communities in wheat fields across the north China plain. Microbiome 6, 27. doi: 10.1186/s40168-018-0409-4 29402331 PMC5799910

[B50] Song Z. Zhang R. H. Fu W. D. Zhang T. Yan J. Zhang G. L. (2017). High-throughput sequencing reveals bacterial community composition in the rhizosphere of the invasive plant *Flaveria bidentis* . Weed Res. 57, 204–211. doi: 10.1111/wre.12250

[B51] Suding K. N. Larson J. R. Thorsos E. Steltzer H. Bowman W. D. (2004). Species effects on resource supply rates: Do they influence competitive interactions? Plant Ecol. 175, 47–58. doi: 10.1023/B:VEGE.0000048093.92118.27

[B52] Sun Y. Y. Zhang Q. X. Zhao Y. P. Diao Y. H. Gui F. R. Yang G. Q. (2020). Beneficial rhizobacterium provides positive plant-soil feedback effects to *Ageratina adenophora* . J. Integr. Agric. 19, 2–10. doi: 10.1016/S2095-3119(20)63234-8

[B53] Tariq M. Hameed S. Malik K. A. Hafeez F. Y. (2007). Plant root associated bacteria for zinc mobilization in rice. Pak J. Bot. 39, 245–253. doi: 10.1111/j.1471-4159.1986.tb13104.x

[B54] te Beest M. Esler K. J. Richardson D. M. (2015). Linking functional traits to impacts of invasive plant species: a case study. Plant Ecol. 216, 293–305. doi: 10.1007/s11258-014-0437-5

[B55] Wang Z. Yu Z. X. Solanki M. K. Yang L. T. Xing Y. X. Dong D. F.. (2020). Diversity of sugarcane root-associated endophytic bacillus and their activities in enhancing plant growth. J. Appl. Microbiol. 128, 814–827. doi: 10.1111/jam.14512 31710757

[B56] Weidenhamer J. D. Callaway R. M. (2010). Direct and indirect effects of invasive plants on soil chemistry and ecosystem function. J. Chem. Ecol. 36, 59–69. doi: 10.1007/s10886-009-9735-0 20077127

[B57] Whittaker R. H. (1972). Evolution and measurement of species diversity. Taxon. 21, 213–251. doi: 10.2307/1218190

[B58] Wu A. P. Liu L. Qi L. Y. Zhong W. Liang Y. S. Chen F. L.. (2019). Rapid nitrogen and phosphorus homeostasis transformation in *Ageratina adenophora* during invasion. Weed Res. 59, 387–395. doi: 10.1111/wre.12375

[B59] Xu H. G. Qiang S. (2004). Catalog of invasive species in China (Beijing: China environmental science press).

[B60] Zhang F. J. Li Q. Yerger E. H. Chen X. Shi Q. Wan F. H. (2018). AM fungi facilitate the competitive growth of two invasive plant species, *Ambrosia artemisiifolia* and *Bidens pilosa* . Mycorrhiza 28, 703–715. doi: 10.1007/s00572-018-0866-4 30220052

[B61] Zhang C. Niu D. Zhang X. L. Fu H. (2022). Plant functional traits shape growth rate for xerophytic shrubs. Plant Biol. 24, 205–214. doi: 10.1111/plb.13317 34693599

[B62] Zhang N. Wang D. D. Liu Y. P. Li S. Q. Shen Q. R. Zhang R. F. (2014). Effects of different plant root exudates and their organic acid components on chemotaxis, biofilm formation and colonization by beneficial rhizosphere-associated bacterial strains. Plant Soil 374, 689–700. doi: 10.1007/s00572-018-0866-4

[B63] Zhou Y. Staver A. C. (2019). Enhanced activity of soil nutrient-releasing enzymes after plant invasion: a meta-analysis. Ecology 100, e02830. doi: 10.1002/ecy.2830 31323119

[B64] Zhu X. Z. Li Y. P. Feng Y. L. Ma K. P. (2017). Response of soil bacterial communities to secondary compounds released from *Eupatorium adenophorum* . Biol. Invasions 19, 1471–1481. doi: 10.1007/s10530-017-1371-y

