# Supplementary figures and images for: Bacillus benefits the competitive growth of Ambrosia artemisiifolia by increasing available nutrient levels
Source: Front Plant Sci. 2023 Jan 12;13:1069016. doi: 10.3389/fpls.2022.1069016 (PMC9879014; doi:10.3389/fpls.2022.1069016)

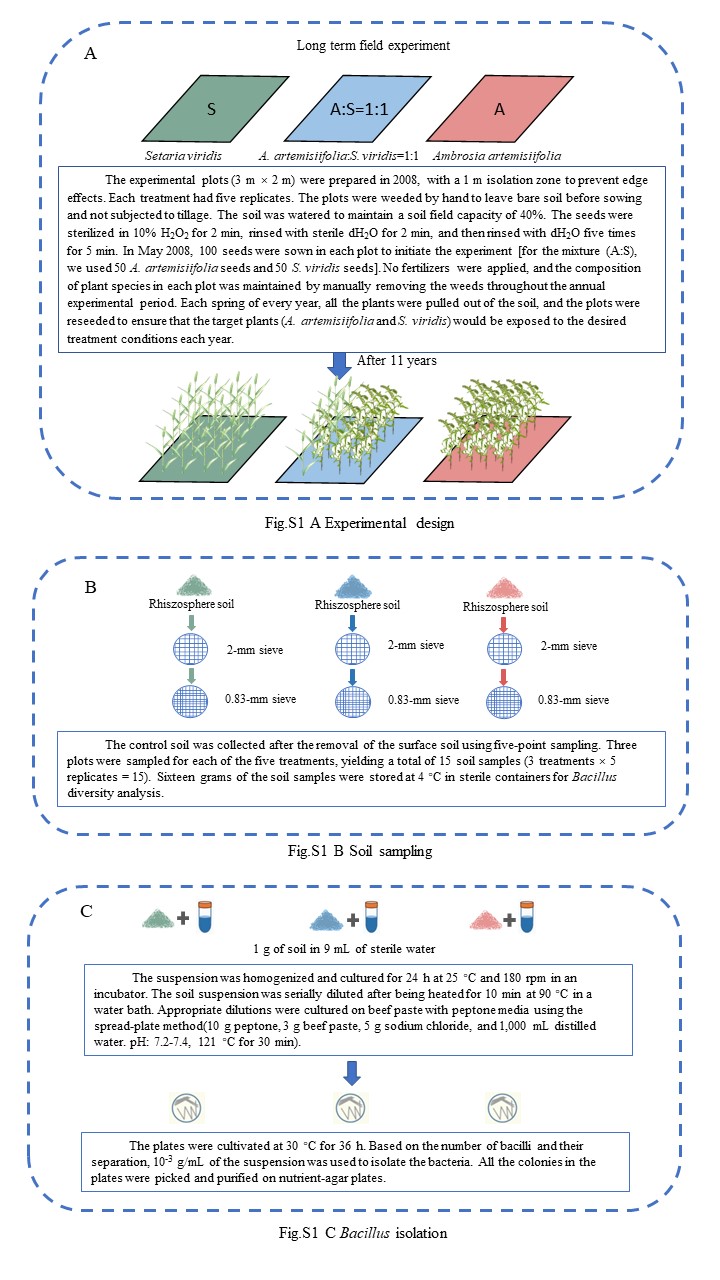

Supplement: Supplementary file 1 [file Image_1.jpeg]

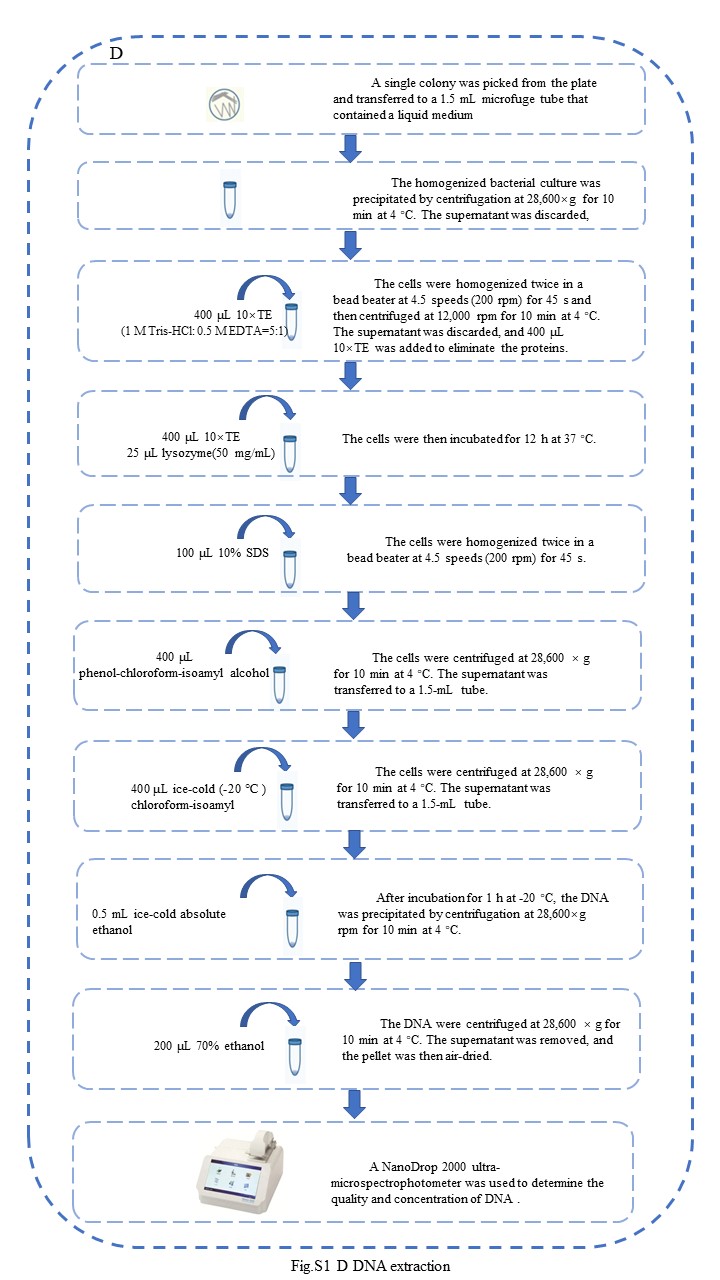

Supplement: Supplementary file 2 [file Image_2.jpeg]

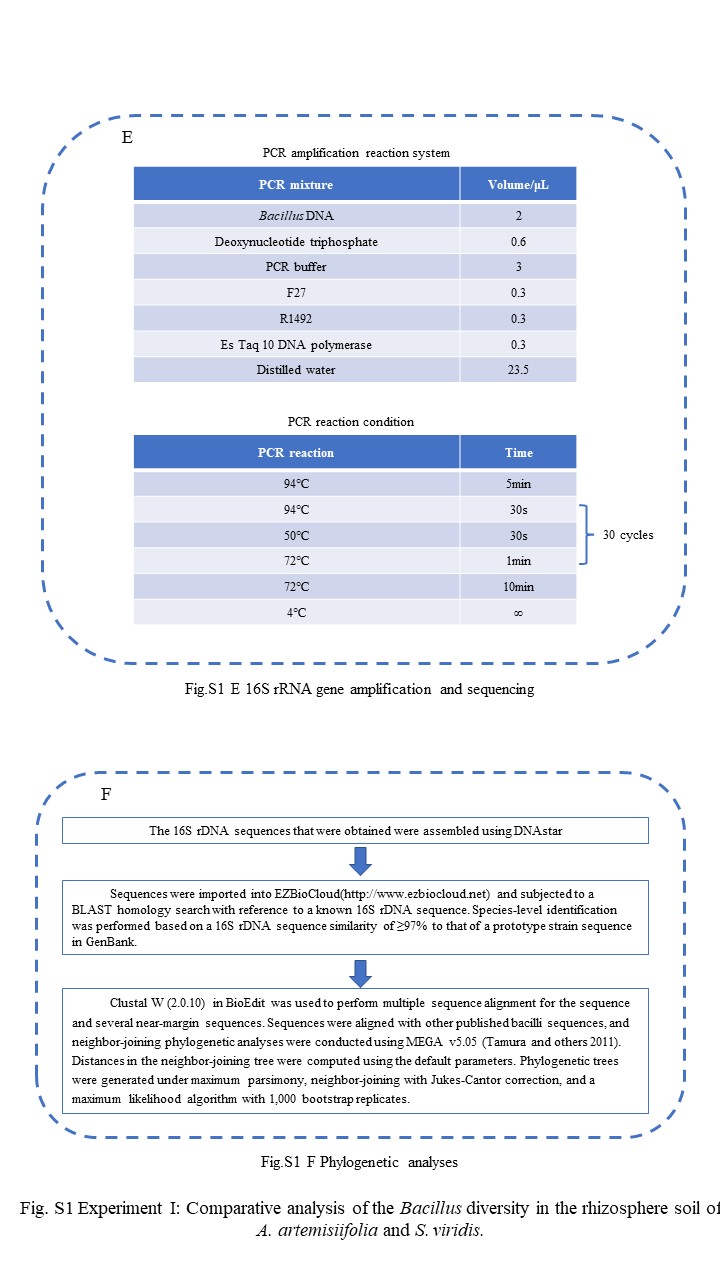

Supplement: Supplementary file 3 [file Image_3.jpeg]

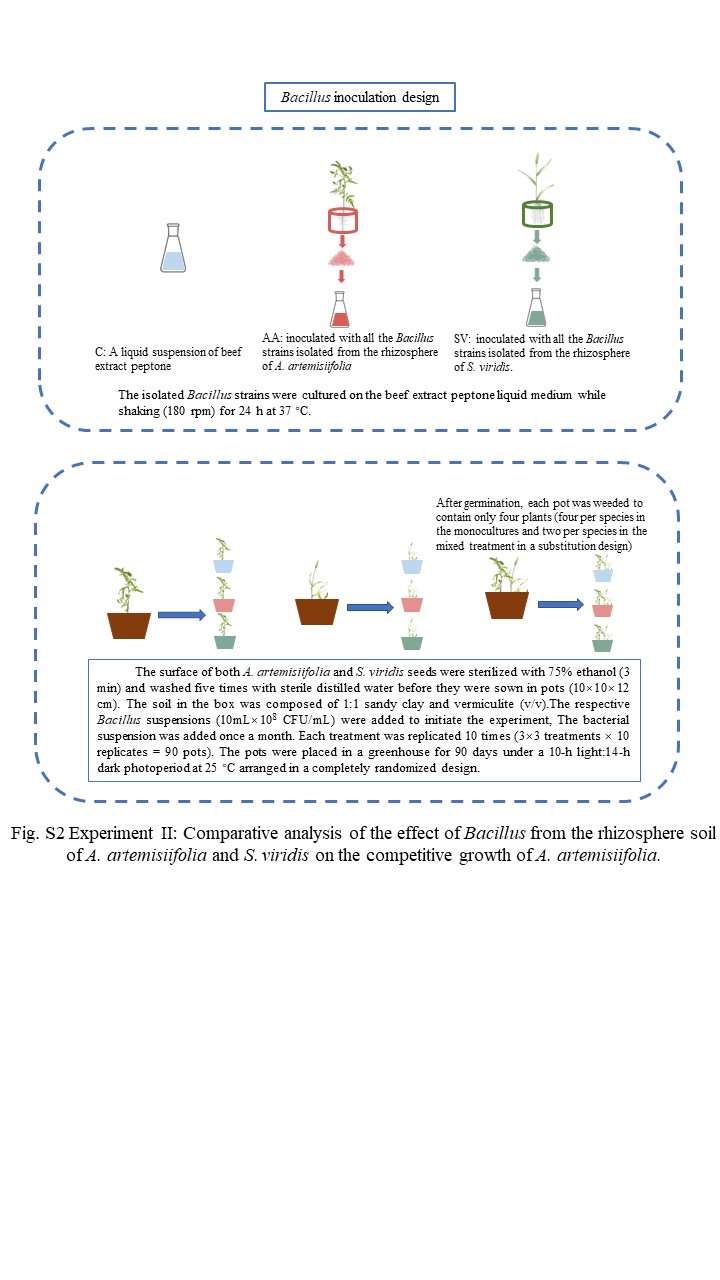

Supplement: Supplementary file 4 [file Image_4.jpeg]

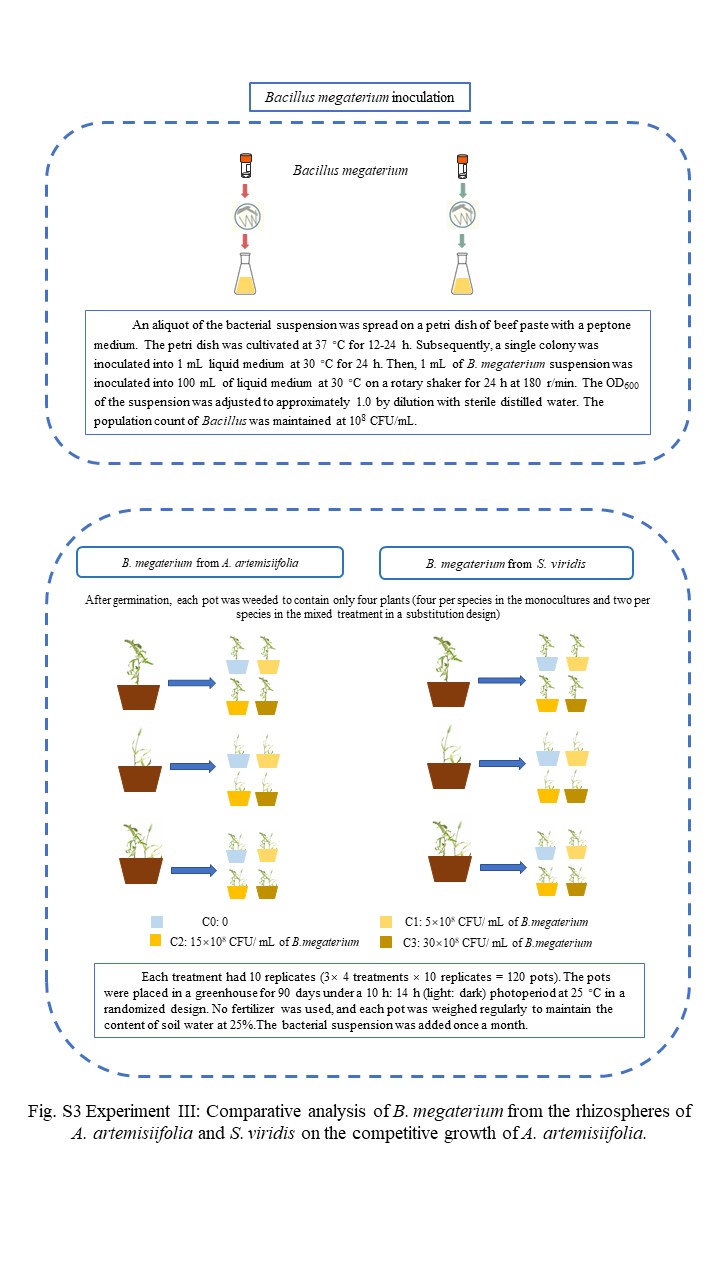

Supplement: Supplementary file 5 [file Image_5.jpeg]

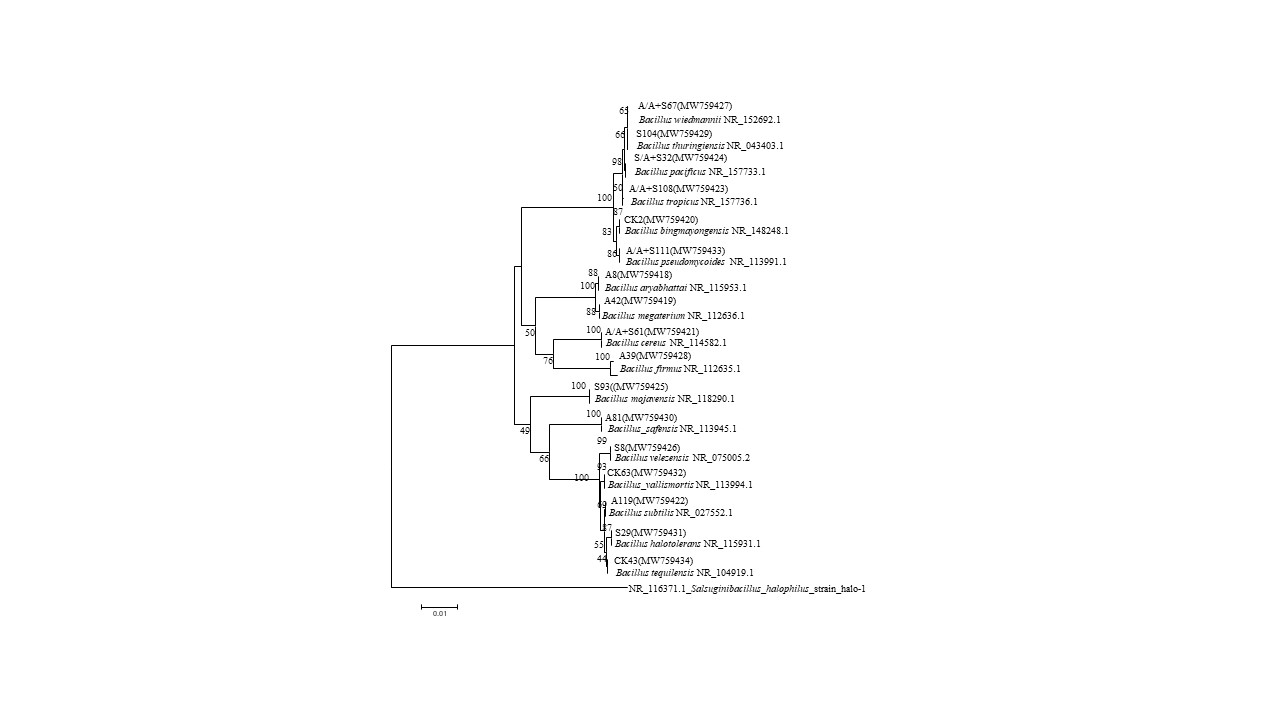

Supplement: Supplementary file 6 [file Image_6.jpeg]

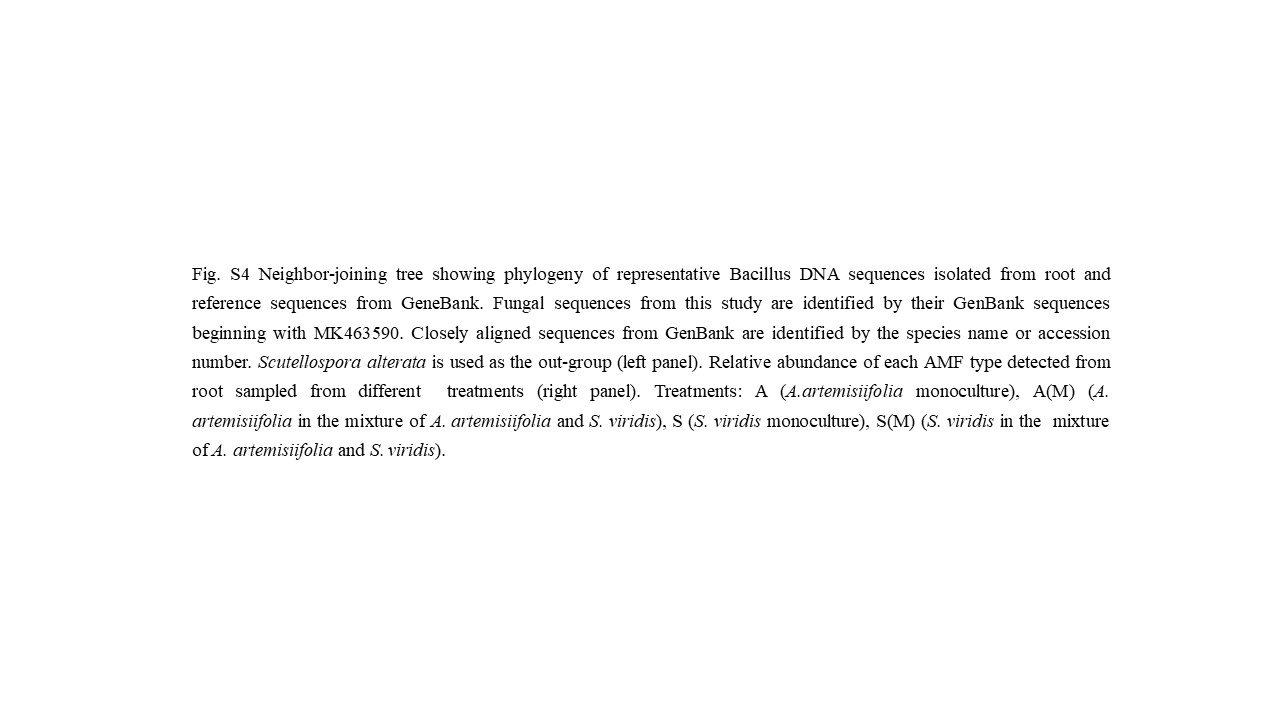

Supplement: Supplementary file 7 [file Image_7.jpeg]

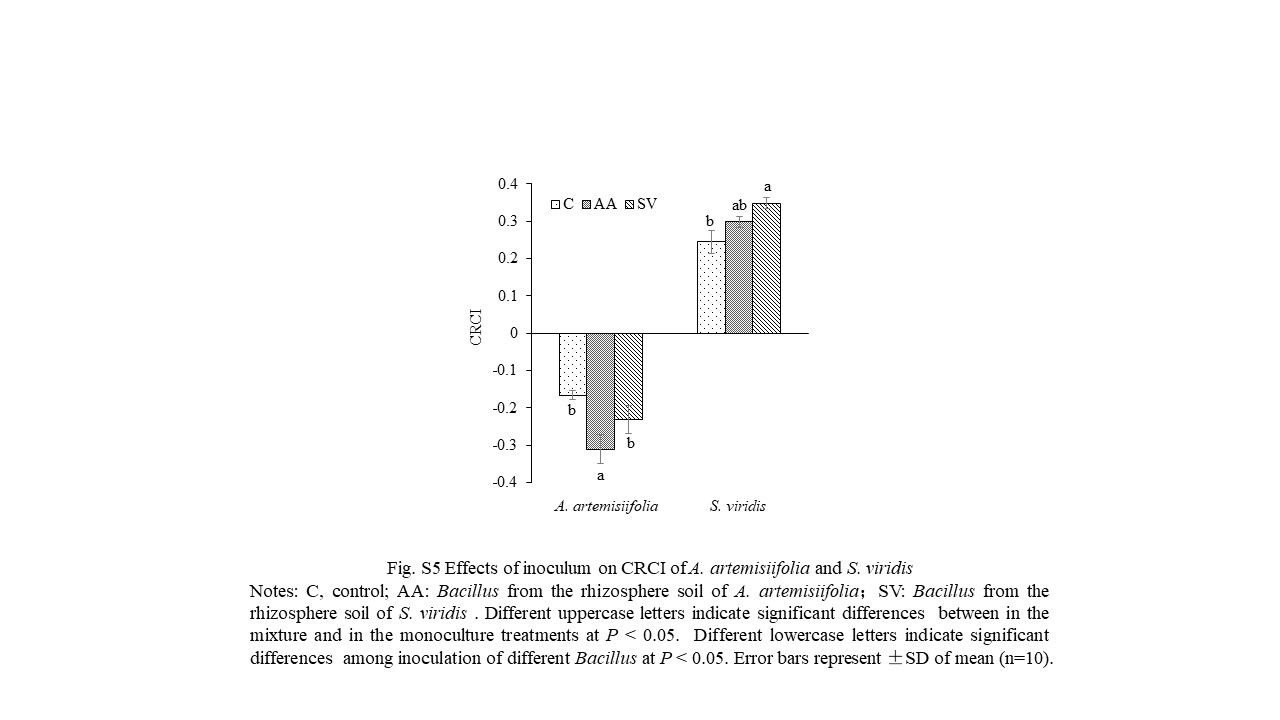

Supplement: Supplementary file 8 [file Image_8.jpeg]

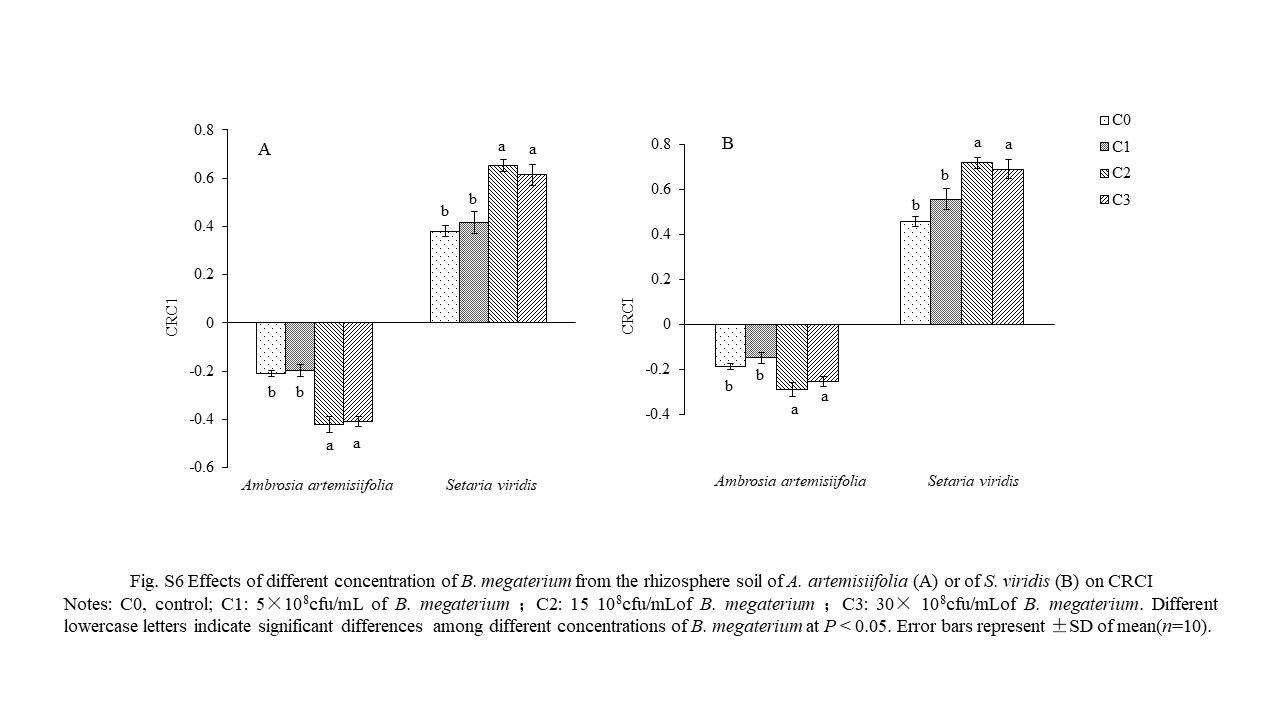

Supplement: Supplementary file 9 [file Image_9.jpeg]
