# Supplementary material for: Bacillus benefits the competitive growth of Ambrosia artemisiifolia by increasing available nutrient levels
Source: Front Plant Sci. 2023 Jan 12;13:1069016. doi: 10.3389/fpls.2022.1069016 (PMC9879014; doi:10.3389/fpls.2022.1069016)
Supplement: Supplementary file 10 [file Table_1.docx]

Table S1 Summary of *Bacillus* species from each treatment soil

| species | CK | A | S | A/A:S | S/A:S |
| --- | --- | --- | --- | --- | --- |
| *Bacillus aryabhattai* | 54.33±2.08c | 25.33±0.58e | 50.33±2.52d | 84.33±1.53b | 90.33±2.08a |
| *Bacillus megaterium* | 42.33±3.21b | 87.00±3.61a | 30.00±2.00c | 24.00±1.00d | 16.33±1.53e |
| *Bacillus bingmayongensis* | 12.33±1.53a | 0.33±0.58b | 0.00±0.00b | 0.00±0.00b | 0.00±0.00b |
| *Bacillus cereus* | 11.67±2.08a | 1.33±0.58c | 10.67±1.15a | 7.00±1.00b | 0.00±0.00c |
| *Bacillus subtilis* | 5.33±0.58a | 1.67±0.58b | 5.00±1.00a | 2.33±0.58b | 1.67±0.58b |
| *Bacillus tropicus* | 0.00±0.00b | 0.00±0.00b | 0.00±0.00b | 1.67±0.58a | 0.00±0.00b |
| *Bacillus pcificus* | 5.00±1.00a | 0.00±0.00c | 0.00±0.00c | 1.67±0.58b | 0.67±0.58bc |
| *Bacillus mojavensis* | 0.00±0.00c | 0.00±0.00c | 4.67±0.58a | 0.00±0.00c | 1.00±0.00b |
| *Bacillus velezensis* | 0.00±0.00b | 0.00±0.00b | 8.00±1.00a | 0.00±0.00b | 0.67±0.58b |
| *Bacillus wiedmannii* | 7.67±1.53a | 0.00±0.00c | 2.00±1.00b | 1.33±0.58bc | 0.00±0.00c |
| *Bacillus firmus* | 0.00±0.00b | 0.67±0.58a | 0.00±0.00b | 0.00±0.00b | 0.00±0.00b |
| *Bacillus thuringiensis* | 3.00±1.00b | 2.00±1.00b | 0.00±0.00c | 5.33±0.58a | 0.00±0.00c |
| *Bacillus safensis* | 0.00±0.00b | 1.67±0.58a | 0.00±0.00b | 0.00±0.00b | 0.00±0.00b |
| *Bacillus halotolerans* | 3.33±0.58b | 0.00±0.00c | 4.67±0.58a | 0.00±0.00c | 0.00±0.00c |
| *Bacillus vallismortis* | 2.67±0.58a | 0.00±0.00b | 2.67±0.58a | 0.00±0.00b | 0.00±0.00b |
| *Bacillus proteolyticus* | 6.00±1.00a | 0.00±0.00c | 2.67±0.58b | 2.33±0.58b | 0.00±0.00c |
| *Bacillus pseudomycoides* | 0.00±0.00b | 0.00±0.00b | 0.00±0.00b | 5.33±0.58a | 0.00±0.00b |
| *Bacillus tequilensis* | 1.00±0.00a | 0.00±0.00b | 0.00±0.00b | 0.00±0.00b | 0.00±0.00b |
| Mean value of the number of *Bacillus* | 51 | 41 | 40.67 | 45.67 | 37.33 |
| Total number of *Bacillus* species | 12 | 7 | 10 | 11 | 6 |

Treatments: CK (bare soil), A (*A.artemisiifolia* monoculture), A/A:S (*A. artemisiifolia* in the mixture of *A. artemisiifolia* and *S. viridis*), S (*S. viridis* monoculture), S/A:S (*S. viridis* in the mixture of *A. artemisiifolia* and *S. viridis*). Different lowercase letters indicate significant differences of the same indicator among different treatments at *P* < 0.05. Error bars represent ±1SE of mean (n = 3).

Table S2 Two-way ANOVA P-value for biomass, C, P, and N content responses of *A. artemisiifolia* and *S. viridis* to inoculation with *B megaterium* (BM) from *A. artemisiifolia*, and plant competition (PC)

| Treatments | Effect | df | Biomass | C content | P content | N content | available phosphorus | available nitrogen |
| --- | --- | --- | --- | --- | --- | --- | --- | --- |
|  | PC | 1 | <0.001 | <0.001 | <0.001 | <0.001 | <0.001 | <0.001 |
| *A. artemisiifolia* | BM | 3 | <0.001 | <0.001 | <0.001 | <0.001 | <0.001 | <0.001 |
|  | PC*BM | 3 | <0.001 | 0.987 | 0.096 | 0.01 | 0.983 | 0.009 |
|  | PC | 1 | <0.001 | <0.001 | <0.001 | <0.001 | <0.001 | 0.004 |
| *S. viridis* | BM | 3 | <0.001 | <0.001 | <0.001 | <0.001 | <0.001 | <0.001 |
|  | PC*BM | 3 | <0.001 | 0.99 | 0.965 | 0.136 | 0.01 | 0.057 |

Table S3 Two-way ANOVA P-value for biomass, C, P, and N content responses of *A. artemisiifolia* and *S. viridis* to inoculation with *B megaterium* (BM) from *S. viridis*, and plant competition (PC)

| Treatments | Effect | df | Biomass | C content | P content | N content | available phosphorus | available nitrogen |
| --- | --- | --- | --- | --- | --- | --- | --- | --- |
|  | PC | 1 | <0.001 | <0.001 | <0.001 | <0.001 | 0.055 | 0.030 |
| *A. artemisiifolia* | BM | 3 | <0.001 | <0.001 | <0.001 | <0.001 | 0.399 | 0.424 |
|  | PC*BM | 3 | <0.001 | 0.686 | 0.928 | <0.001 | 0.454 | 0.105 |
|  | PC | 1 | <0.001 | <0.001 | <0.001 | <0.001 | 0.087 | 0.002 |
| *S. viridis* | BM | 3 | <0.001 | <0.001 | <0.001 | <0.001 | 0.222 | <0.001 |
|  | PC*BM | 3 | <0.001 | 0.876 | 0.985 | 0.275 | 0.311 | 0.977 |

Table S4 Correlation between the concentration of *B megaterium* from *A. artemisiifolia* and plant growth indicators

| Treatments | | | biomass | C content | P content | N content | available phosphorus | available nitrogen |
| --- | --- | --- | --- | --- | --- | --- | --- | --- |
| *A. artemisiifolia* | Mon | C0 | 0.921* | 0.908** | 0.944* | 0.971** | 0.987** | 0.938* |
|  |  | C1  C2  C3 | 0.889*  0.961**  0.893* | 0.901*  0.914*  0.927* | 0.896*  0.887*  0.949* | 0.950*  0.932*  0.901* | 0.919*  0.938*  0.967** | 0.956*  0.923*  0.901** |
|  | Mix | C0 | 0.954* | 0.849 | 0.897* | 0.988* | 0.942* | 0.966* |
|  |  | C1  C2  C3 | 0.899*  0.973**  0.948* | 0.937*  0.971**  0.979** | 0.916*  0.920*  0.906* | 0.893*  0.971**  0.948* | 0.972*  0.966**  0.937* | 0.889*  0.952*  0.951* |
| *S. viridis* | Mon | C0 | 0.910* | 0.922* | 0.922* | 0.960* | 0.582 | 0.962 |
|  |  | C1  C2  C3 | 0.977*  0.898*  0.910* | 0.978*  0.879*  0.934* | 0.306  0.814  0.948* | -0.515  0.739  0.794 | -0.303  0.950*  -0.282 | 0.699  0.775  0.378 |
|  | Mix | C0 | 0.850 | 0.849 | 0.939* | 0.329 | 0.870 | -0.022 |
|  |  | C1  C2  C3 | 0.849  0.868  0.976* | 0.699  0.871  0.581 | 0.133  0.468  0.941* | 0.948*  0.649  0.314 | 0.972*  0.726  0.937* | 0.097  0.952  0.445 |

Table S5 Correlation between the concentration of *B megaterium* from *S. viridis* and plant growth indicators

| Treatments | | | biomass | C content | P content | N content | available phosphorus | available nitrogen |
| --- | --- | --- | --- | --- | --- | --- | --- | --- |
| *A. artemisiifolia* | Mon | C0 | 0.988* | 0.975** | 0.764 | 0.856 | 0.982** | 0.913** |
|  |  | C1  C2  C3 | 0.996**  0.763  0.733 | 0.983**  0.788  0.998** | 0.757  0.739  0.985** | 0.961*  0.958*  0.777 | 0.939*  0.761  0.955* | 0.965*  0.830  0.951* |
|  | Mix | C0 | 0.939* | 0.934* | 0.927* | 0.981** | 0.971** | 0.997** |
|  |  | C1  C2  C3 | 0.930*  0.902*  0.864 | 0.971**  0.821  0.953* | 0.974**  0.863  0.850 | 0.935*  0.946*  0.954* | 0.939*  0.943*  0.781 | 0.869  0.870  0.651 |
| *S. viridis* | Mon | C0 | 0.954* | 0.968** | 0.924* | 0.950* | 0.872 | 0.973** |
|  |  | C1  C2  C3 | 0.944*  0.885*  0.961** | 0.968**  0.927*  0.911* | 0.944*  0.882*  0.925* | 0.885*  0.930*  0.892* | 0.954*  0.807  0.963** | 0.862  0.968**  0.994 |
|  | Mix | C0 | 0.971** | 0.956 | 0.682 | 0.822 | 0.971** | 0.997* |
|  |  | C1  C2  C3 | 0.961**  0.956*  0.966** | 0.815  0.852  0.815 | 0.746  0.972**  0.989** | 0.912*  0.925*  0.881* | 0.939*  0.943*  0.781 | 0.869  0.860  0.651 |
